# Photoluminescence Properties of Silver(I) Complexes: From Extremely Long-Lived Phosphorescence to Ultrashort-Lived TADF

**DOI:** 10.1021/acs.inorgchem.6c00401

**Published:** 2026-05-18

**Authors:** Hartmut Yersin, Marsel Z. Shafikov, Uwe Monkowius, Ruslan Ramazanov, Rashid Valiev

**Affiliations:** † Institute of Physical Chemistry, 9147 University of Regensburg, 93053 Regensburg, Germany; ‡ Department of Chemistry, Texas A&M University, College Station, Texas 77842-3012, United States; § School of Education, 27266 Johannes Kepler University Linz, 4040 Linz, Austria; ∥ Institute of Inorganic Chemistry, Johannes Kepler University Linz, 4040 Linz, Austria; ⊥ Department of Chemistry, 3835 University of Helsinki, 00014 Helsinki, Finland

## Abstract

We review Ag­(I) complexes with organic ligands that show ambient-temperature phosphorescence or thermally activated delayed fluorescence (TADF). Criteria for the assignment of the emission character are given. Two materials are highlighted: [Ag­(dmp)­(DPEPhos)]­PF_6_ **A** (dmp: 2,9-dimethyl-1,10-phenanthroline, DPEPhos: bis­[(2-diphenylphosphino)­phenyl]­ether), featuring an exceptionally long decay time of τ­(phos) = 110 ms at Φ_PL_(phos) ≈50%, shows phenanthroline ligand-centered ^3^LC → S_0_ phosphorescence. The resulting large Δ*E*(S_1_–T_1_) gap (640 meV) inhibits the efficient thermal population of the S_1_ state. In contrast, the rationally modified Ag­(dbp)­(P_2_-nCB) **B** (dbp: 2,9-dibutyl-1,10-phenanthroline, P_2_-nCB: strongly electron-donating diphosphine ligand comprising a negatively charged *nido*-carborane cage) features low-lying states of (ligand + metal)-to-ligand charge transfer (^1,3^(L + M)­L’CT) character. This reduces Δ*E*(S_1_–T_1_) to 80 meV, enabling fast and bright ambient-temperature TADF (radiative decay time: 1.4 μs at Φ_PL_(TADF) = 100%) with the decay time of more than 5 orders of magnitude shorter than that of **A**. The radiative properties of both compounds are outstanding compared to other pseudotetrahedrally coordinated metal complexes. In a second part, we extend the reviewed work by investigating both compounds by detailed DFT and TD-DFT calculations, including spin–orbit coupling. The results reflect the experimental trends. **A** is a potential highly sensitive oxygen sensor, while **B** might represent a new type of efficient OLED emitter material.

## Graphical abstract

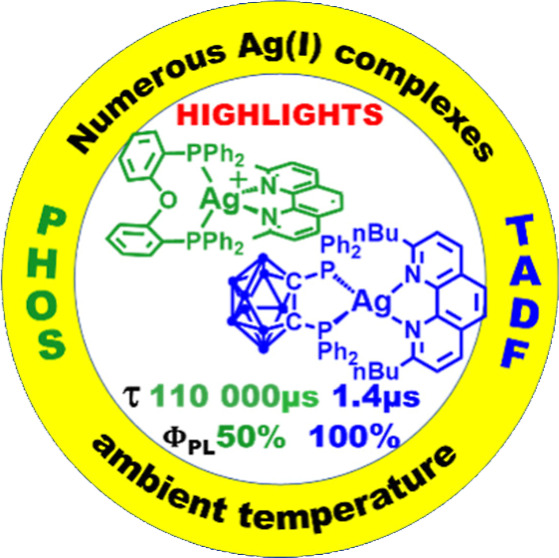

## 1 Introduction

Photoluminescent properties of Ag­(I) complexes with organic ligands attracted pronounced interest only recently, in contrast to extensive research on homologous Cu­(I) compounds. This is due to the fact that these latter ones are typically characterized by low-lying excited states dominated by metal (3d)-to-ligand charge transfer character (^1,3^MLCT).^1–4^ Usually, the corresponding singlet S_1_ to triplet T_1_ energy gap Δ*E*(S_1_–T_1_) = Δ*E*(^1^MLCT–^3^MLCT) is small enough, typically around 1000 cm^–1^ (125 meV) or smaller, to allow for efficient T_1_ → S_1_ up-intersystem crossing (up-ISC) at ambient temperature.^4^ Frequently, up-ISC is also termed reverse ISC (RISC). As a consequence, relatively short-lived thermally activated delayed fluorescence (TADF), normally decaying in several microseconds, dominates the emission behavior.^4–6^ In particular, efficient thermal population of the excited singlet states endows the TADF compounds to harvest and efficiently utilize both types of excitons, singlets and triplets, formed in electroluminescent devices (OLEDs). Accordingly, 100% internal electroluminescence efficiency becomes possible. This strongly stimulated research activities in the field of Cu­(I)-complex based research.^7–9^ It is noted that originally, the TADF effect applied in OLEDs was designated as “singlet harvesting effect”.^7,8,10^ On the other hand, as the ionization energy of the Ag­(I) ion (second ionization energy) is larger by 1.2 eV than that of the Cu­(I) ion,^11^ it is indicated that for homologous complexes, the Ag­(I) 4d-orbitals lie at significantly deeper energy than the Cu­(I) 3d-orbitals. As a consequence, the lowest excited states of most of the early synthesized silver­(I) complexes do not feature low-lying ^1,3^MLCT states as those of homologous Cu­(I) complexes but instead are of ligand-centered ^1,3^LC character resulting in a large energy gap Δ*E*(S_1_–T_1_) = Δ*E*(^1^LC–^3^LC) of the order of 1 eV (8066 cm^–1^). Thus, at first sight, the occurrence of TADF is improbable for Ag­(I) complexes. Accordingly, in early studies, the emission observed was frequently assigned as ^3^LC → S_0_ phosphorescence with slow radiative rate and frequently with low emission quantum yield. Therefore, research interest in photoluminescent metalorganic-silver­(I) complexes was less pronounced than in Cu­(I) materials.

However, within the past decade, it was recognized that molecular design with judicious ligand functionalization can afford Ag­(I) complexes that, on the one hand, feature high phosphorescence quantum yield and, on the other hand, very efficient and fast TADF. Accordingly, investigation of emission properties of Ag­(I) compounds became increasingly more attractive. Hence, in the scope of the present study in Section 2, we will give a short review on design strategies of Ag­(I) complexes and summarize emission properties, including assignment of the low-energy transitions. In Section 3, we will focus on the emission behavior of the specific compound [Ag­(dmp)­(DPEPhos)]­PF_6_, compound **A** (Figure 1). It represents an outstanding material that shows extremely long-lived ligand-centered phosphorescence decay time (>100 ms) at high emission quantum yield (Φ_PL_ = 50%).^12^ Potentially, this compound is well-suited as optical oxygen sensor material.

**Figure 1:**
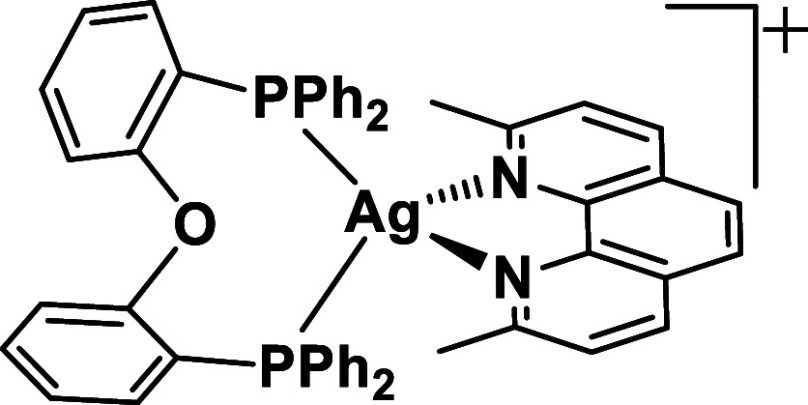
[Ag­(dmp)­(DPEPhos)]^+^, cation in compound **A**. Milestone material that shows extremely long-lived phosphorescence at ambient temperature ([Ag­(dmp)­(DPEPhos)]­PF_6_ in PMMA: τ = 110 ms, Φ_PL_ = 50%).^12^ Frequently, DPEPhos is also abbreviated as POP or dpep.

Subsequently, in Section 4, we present chemical design rules for modification of such complexes showing ligand-centered phosphoresce into complexes with lowest excited states of dominant ligand-to-ligand charge transfer (LL’CT) character that also involve some 4d-metal contribution. For illustration of the changes of orbital energies connected with such chemical modifications, Figure 6 is shown below. Thus, in this case of low-lying ^1,3^LL’CT states, occurrence of TADF emission is expected as the energy gap Δ*E*(S_1_–T_1_) decreases drastically compared to the ^1,3^LC situation. Explanations are given in Section 4. Indeed, Ag­(dbp)­(P_2_-nCB), compound **B** (Figure 2), represents such an emitter. It even exhibits breakthrough characteristics with an emission quantum yield of Φ_PL_ = 100% and ultrashort-lived TADF with a decay time of τ = 1.4 μs,^13^ thus representing one of a few tetra-coordinated metal complexes featuring TADF that match the radiative rate and quantum yield of *fac*-Ir­(ppy)_3_ (with ppy = 2-phenylpyridine),^14^ the archetypal emitter used in OLEDs. Photophysical properties of compound **B** are characterized in detail in Section 5. Potentially, compound **B** also represents an efficient TADF OLED emitter material.

**Figure 2:**
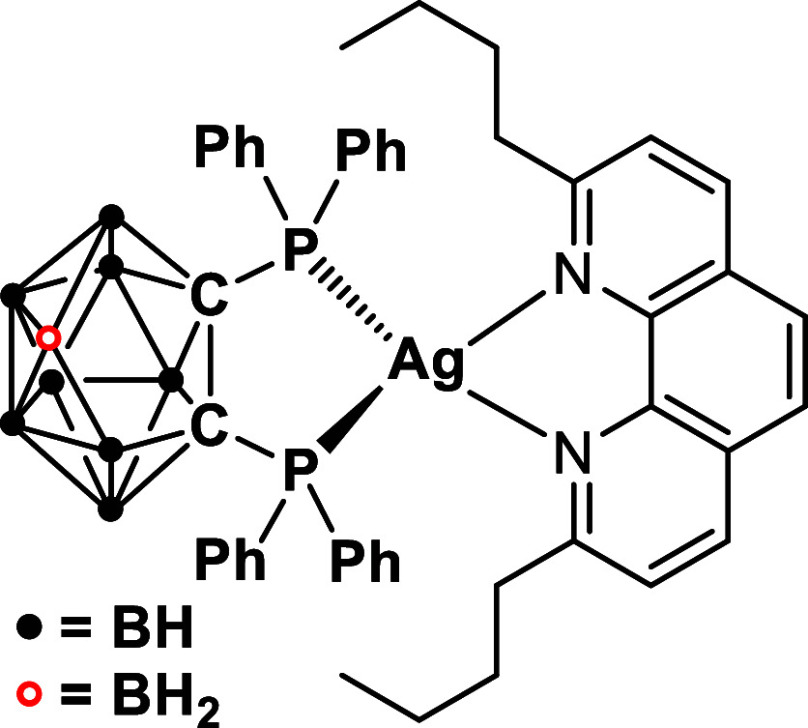
Ag­(dbp)­(P_2_-nCB), compound **B**. Milestone material that shows ultrafast TADF at ambient temperature (neat powder: τ = 1.4 μs, Φ_PL_ = 100%).^13^

**Figure 3:**
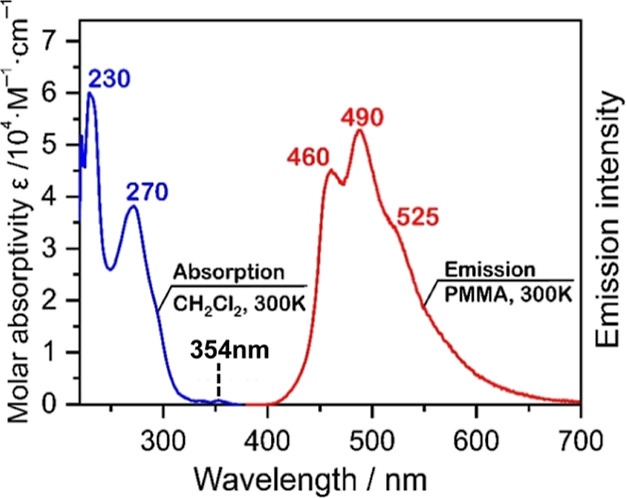
Ambient-temperature absorption and emission spectra of [Ag­(dmp)­(DPEPhos)]­PF_6_, compound **A**. The absorption is measured in CH_2_Cl_2_ solution (*c* ≤ 10^–5^ M), while the emission is recorded from a doped PMMA film with a dopant concentration c ≪ 1 wt %. Adapted with permission from ref 12. Copyright 2019, The Royal Society of Chemistry.

**Figure 4:**
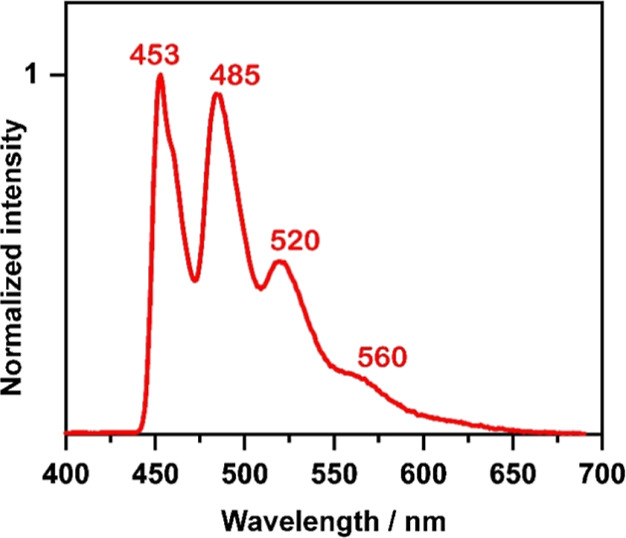
Emission spectrum of [Ag­(dmp)­(DPEPhos)]­PF_6_, compound **A**, measured of a doped PMMA film at *T* = 77 K, dopant concentration c ≪ 1 wt %. The vibrational progression observed is assigned to ≈1400 cm^–1^ vibrations of dmp. Reproduced with permission from ref 12, Supporting Information. Copyright 2019, The Royal Society of Chemistry.

**Figure 5:**
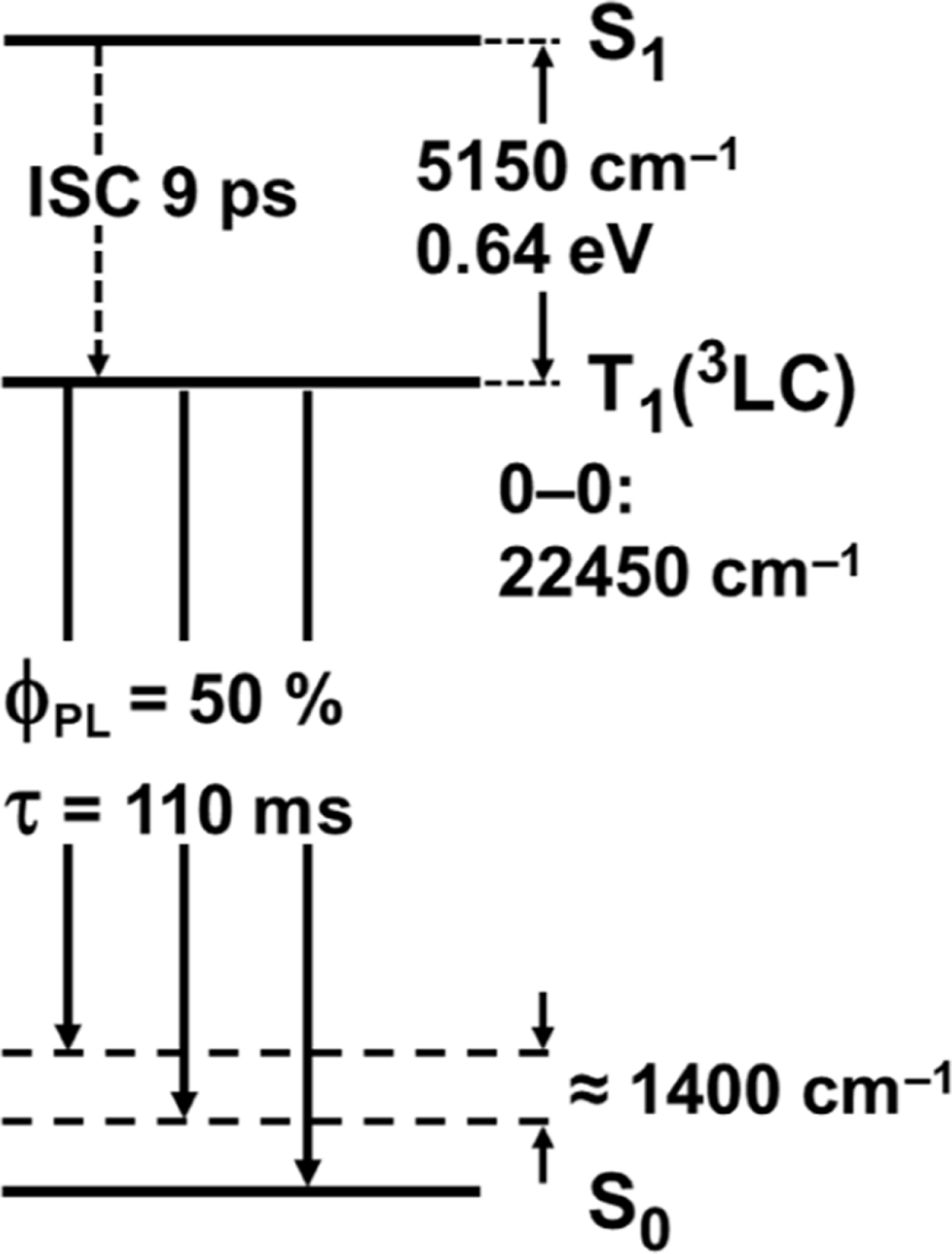
Simplified energy level diagram for [Ag­(dmp)­(DPEPhos)]^+^ (cation in compound **A**) and decay data. The intersystem crossing (ISC) time results from a theoretical estimate calculated for the optimized (relaxed) S_1_ state geometry (Section 6). The 0–0 transition energy is estimated from the 77 K emission spectrum (Figure 4).

In Section 6, we extend the reviewed work by studying both compounds theoretically by density functional theory (DFT) and time-dependent DFT (TD-DFT) methods, including spin–orbit coupling (SOC) effects. Finally, in Section 7, this minireview is concluded.

## 2 Silver(I) Compounds Showing Phosphorescence or TADF

An assignment of the photoluminescence properties of Ag­(I) compounds is not always straightforward. In general, ambient-temperature emission (of different organo-transition silver­(I) complexes) can represent T_1_ → S_0_ ligand-centered phosphorescence or S_1_ → S_0_ delayed fluorescence (TADF).

For identification of ligand-centered (non-CT type) phosphorescence at exclusion of TADF, four criteria are considered:

(i) Relatively slow radiative emission decay rate

Due to energetically deep-lying Ag 4d metal orbitals, the lowest ligand-localized ^3^LC states are energetically far from the states with considerable metal contribution. Hence, the radiative triplet T_1_ to singlet S_0_ transition rates are relatively slow (order of 10^3^ s^–1^ or slower). This is related to the small size of the spin–orbit coupling matrix elements (SOCME). (Details are discussed below in Section 3.) Moreover, in this case, nonradiative quenching becomes competitive and thus can lead to small T_1_ → S_0_ phosphorescence quantum yield, if no specific molecular design approach, such as molecular rigidification, is applied.^4^ As shown below, the situation changes distinctly for TADF emitters.

(ii) Observation of vibrational progression in the emission spectra

Occurrence of resolved vibrational structure is typically related to small molecular geometry changes between electronic ground and excited states.^15^ This is frequently valid for LC transitions, in contrast to CT transitions. The latter ones are due to pronounced charge redistributions within the molecule, connected with significant geometry distortions. These usually lead to unresolved spectral structures.

(iii) Spatial localization of molecular orbitals involved in the transition

Theoretical calculations can provide the relevant frontier orbitals, such as HOMO and LUMO, or natural transition orbitals (NTOs). In particular, spatial localization of the involved orbitals on the same ligand moiety is an indication of an excited state having LC character (Section 6 below).

(iv) Large experimentally determined or calculated energy gap Δ*E*(S_1_–T_1_)

A large energy gap of several hundred meV to around 1 eV indicates a large exchange interaction between the two electrons involved for singlet and triplet state formation. This situation is typical for ligand-centered excited states, as the electrons are confined within the same orbital volume. This is in contrast to ^1,3^CT states featuring significantly reduced overlap of the two unpaired electrons and consequently strongly diminished exchange interaction. In this situation, CT-character singlet and triplet states of the same orbital origin are separated by much smaller energy gaps. Thus, thermally induced population of the higher-energy S_1_ state can become efficient at ambient temperature.

Applying these criteria, we summarize selected literature studies in Table 1. In most cases, several of the criteria are fulfilled. But for two examples (compounds **5** and **6**), only occurrence of vibrational progression in the ambient-temperature emission spectra is reported. Nevertheless, assignment as ^3^LC → S_0_ phosphorescence seems to be justified.^20,21^

**Table 1:**
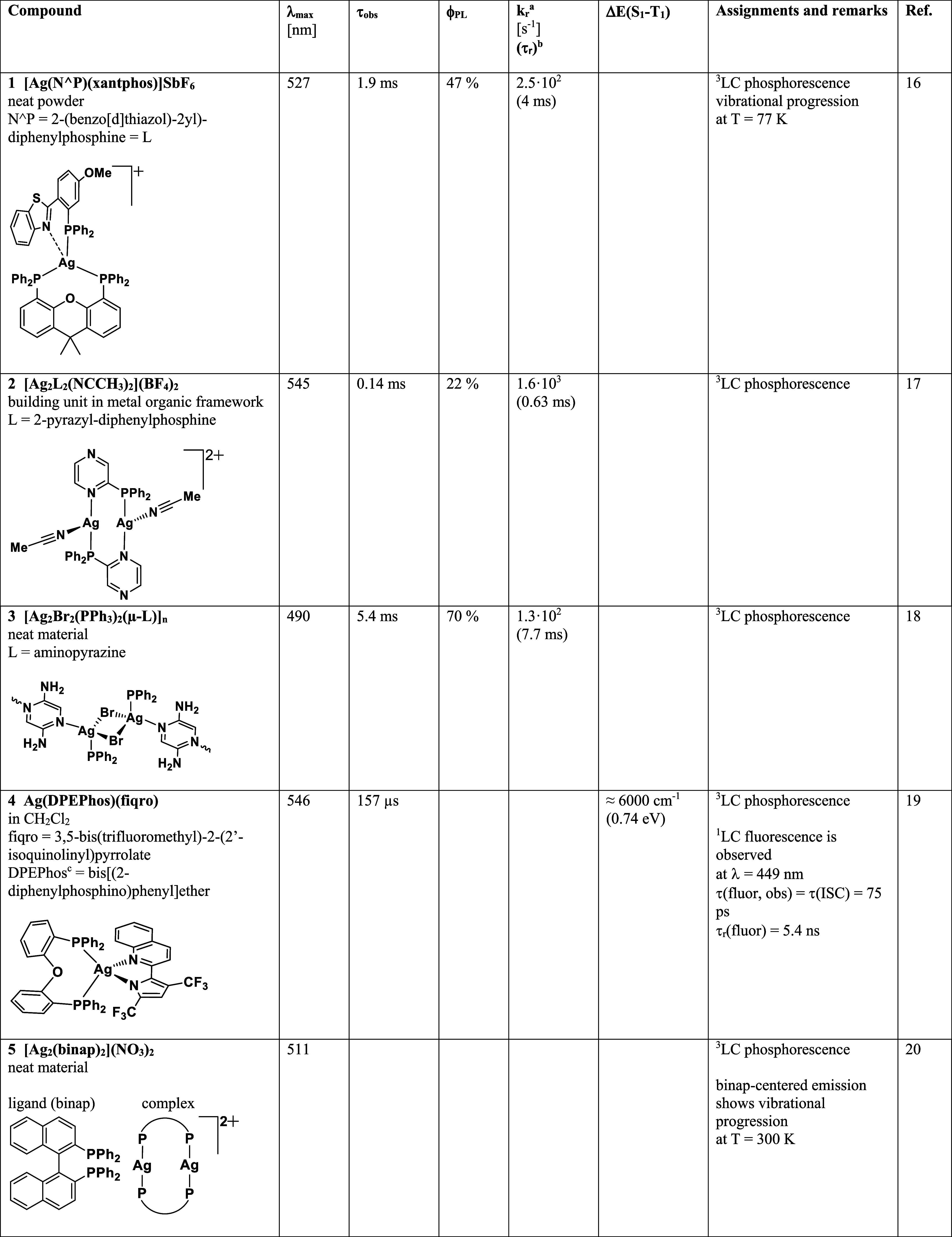

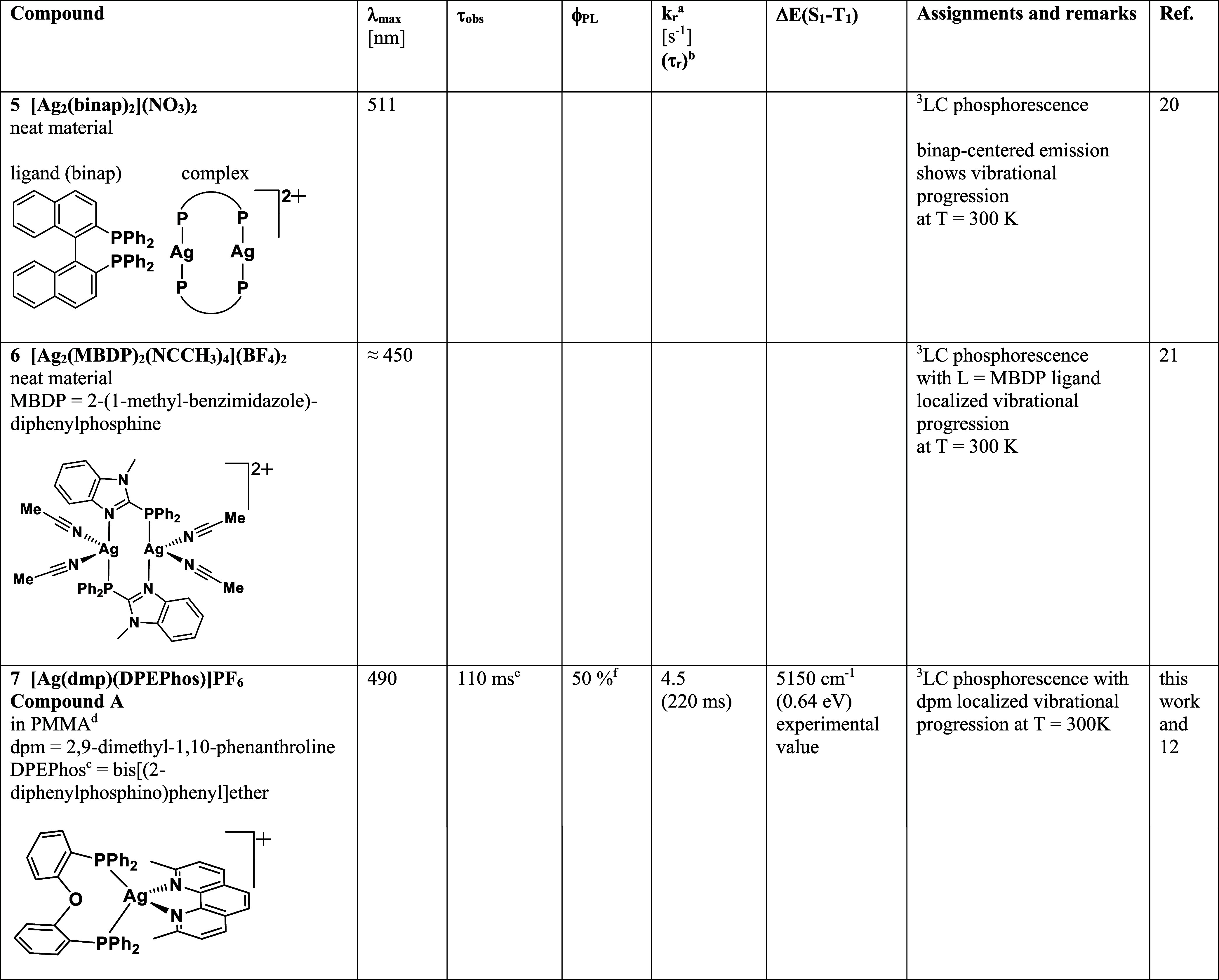
Ag­(I) Complexes That Show Ambient-Temperature Ligand-Centered ^3^LC → S_0_ Phosphorescence

a *k*_r_ = Φ_PL_/τ_obs_.

b τ_r_ = 1/*k*_r_.

c Frequently also abbreviated as POP or dpep.

d PMMA = poly­(methyl methacrylate).

e Measured in vacuum at 10^–6^ bar.

f Measured under N_2_ purging.

Interestingly, compounds **1**,^16^ **2**,^17^ **3**,^18^ and **7** ^12^ exhibit relatively high phosphorescence quantum yield. Presumably, this is related to the rigid packing of the emitting units, which reduces nonradiative processes.^4^ The long radiative emission decay times observed are typical for ^3^LC → S_0_ phosphorescence. (Further explanations are presented in Section 3 based on eq 1.) Compound **4** is characterized by large Δ*E*(S_1_–T_1_) and shows ambient-temperature phosphorescence as well as fluorescence.^19^ The fluorescence decay time of 75 ps is assigned to display the intersystem crossing (ISC) time from S_1_ to T_1_, while the radiative fluorescence decay time is given to τ_r_(fluor) = 5.4 ns. These properties are in line with ligand-centered emission behavior.^19^ For structure **7**, representing the outstanding compound **A**, all four criteria given above for assignment as ^3^LC phosphorescence are fulfilled. The radiative decay rate is as slow as 4.5 s^–1^ (τ_r_ = 220 ms), Δ*E*(S_1_–T_1_) amounts to ≈640 meV (5150 cm^–1^, experimental value), vibrational progression is observed, and calculations show that, for the T_1_ state in its relaxed geometry, hole and electron NTOs are localized on the dmp ligand. Further discussion of the photophysical properties of this material based on experimental and theoretical studies is presented in Sections 3 and 6, respectively.

Assignment of the photoluminescence of silver­(I) compounds as TADF can also be based on clear criteria.

(i) Fast radiative emission decay

In comparison to typical phosphorescence of silver­(I) compounds, the radiative TADF decay rate is one to five orders of magnitude faster, resulting in radiative decay times of around 1 to several 10 μs. Connected with these faster processes, the emission quantum yield Φ_PL_ can become relatively large because nonradiative processes are less competitive in relation to the radiative ones. The reason for the faster radiative decay lies in the TADF process itself as the spin-allowed singlet–singlet transition process is substantially faster than the spin-forbidden T_1_ → S_0_ radiative decay, though RISC processes, important for S_1_ state thermal population, represent a certain bottleneck.^22^

(ii) Small Δ*E*(S_1_–T_1_) gap

For occurrence of TADF, the energy gap Δ*E*(S_1_–T_1_) should not exceed several times the thermal energy at an ambient temperature of 210 cm^–1^ (26 meV), for example, amounting to about 1000 cm^–1^ (125 meV) or smaller to allow for efficient up-ISC processes.^4,6,8–10,13^ As a consequence, the energy gap of efficient TADF compounds is around 1 order of magnitude smaller than that of typical phosphorescent silver­(I) complexes. Under suitable conditions, Δ*E*(S_1_–T_1_) can very roughly be estimated from the transition energy difference between ambient-temperature TADF and low-temperature phosphorescence spectra. A more accurate method for Δ*E*(S_1_–T_1_) determination is based on determination of the thermal activation energy as discussed in Section 5.

(iii) Characteristic temperature behavior of emission

With temperature increase from cryogenic temperature (for example, 77 K or even lower^4^) to ambient temperature, usually, the radiative decay rate increases by one to almost three orders of magnitude. This rate increase is a consequence of the exponential temperature dependence of the RISC processes, which induce thermal activation and emission according to the T_1_ ↔ S_1_ → S_0_ processes. Moreover, usually, the emission spectra show a blue shift (high-energy shift) from low-temperature phosphorescence to ambient-temperature TADF.

(iv) Low-energy donor (D)–acceptor (A) CT transitions

Molecular orbital calculations can give an indication whether the compound exhibits electron-donating (D) and electron-accepting (A) molecular moieties that allow for CT transitions leading to low-energy ^1,3^CT states. In particular, if the D–A orbital overlap is small, as resulting from large D–A separation or almost orthogonal arrangement, small exchange energy and hence small Δ*E*(S_1_–T_1_) gap can result.^4^ As a consequence, efficient RISC is possible, and fast TADF can occur.

(v) Broad and unresolved emission spectra

For completeness, it is mentioned that emission spectra of TADF compounds are usually broad (several thousand cm^–1^) and do not show resolved vibrational structures (progression). This is a consequence of the states’ CT character. Charge transfer occurring upon transition leads to pronounced electron density redistribution followed by geometry changes and hence potential energy surface shifts of the emitting state relative to that of the ground state. In this situation, electronic transitions couple significantly with the vibrations of the complex, local low-energy vibrations, and phonons. This leads to smearing out the spectra. Indeed, all of the TADF spectra observed for Ag­(I) compounds are broad and unstructured.

In Table 2, we summarize selected literature reports on Ag­(I) compounds. All of them (apart from example **11** ^23^) exhibit tetra-coordinated metal centers. All these compounds fulfill most of the criteria given above and thus represent TADF emitters. For completeness, it is mentioned that also two-coordinated compounds of the type of carbene-Ag­(I)-amide can show efficient TADF. Here, however, these are not further discussed as already several comprehensive reports have been published recently.^24–27^

**Table 2:**
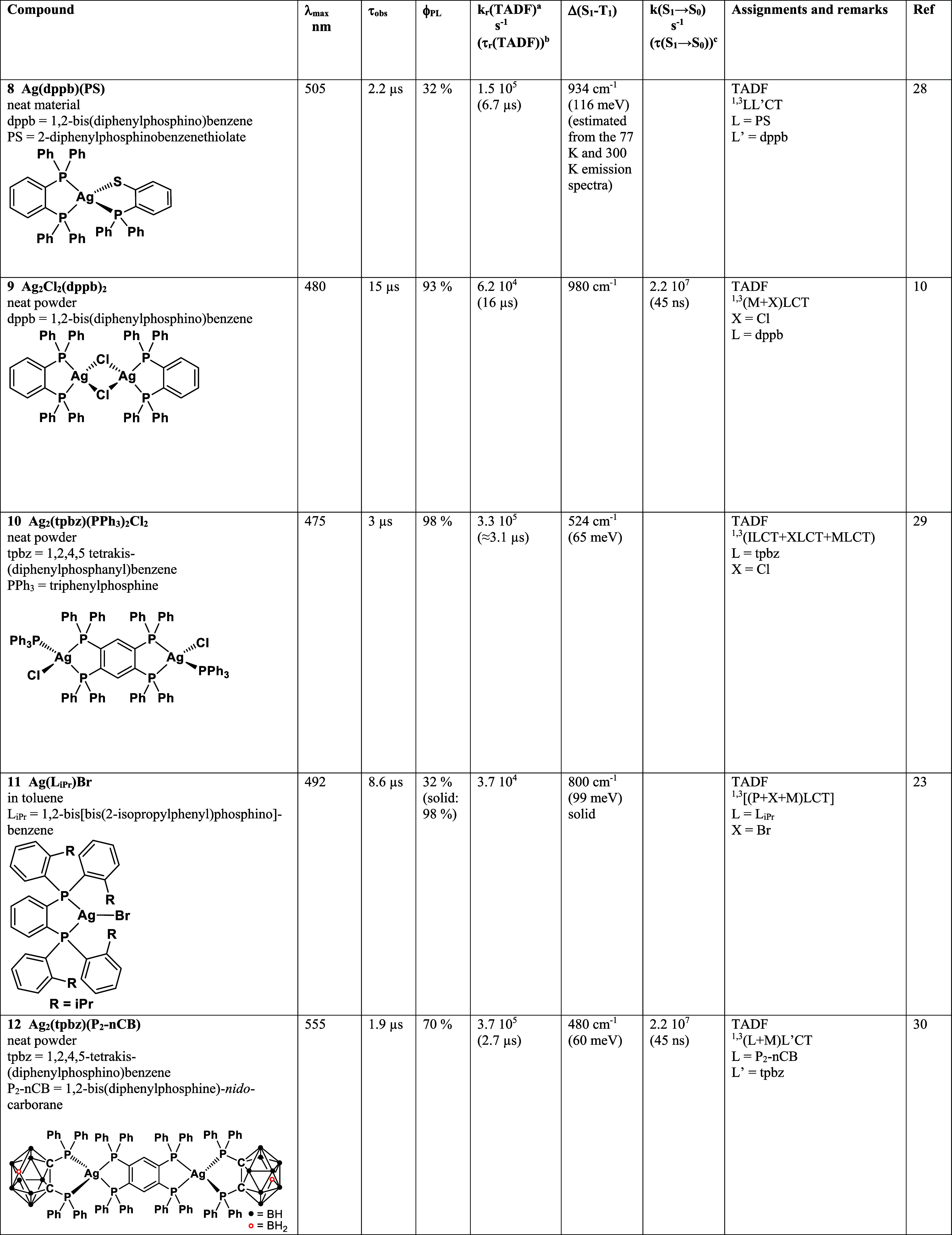

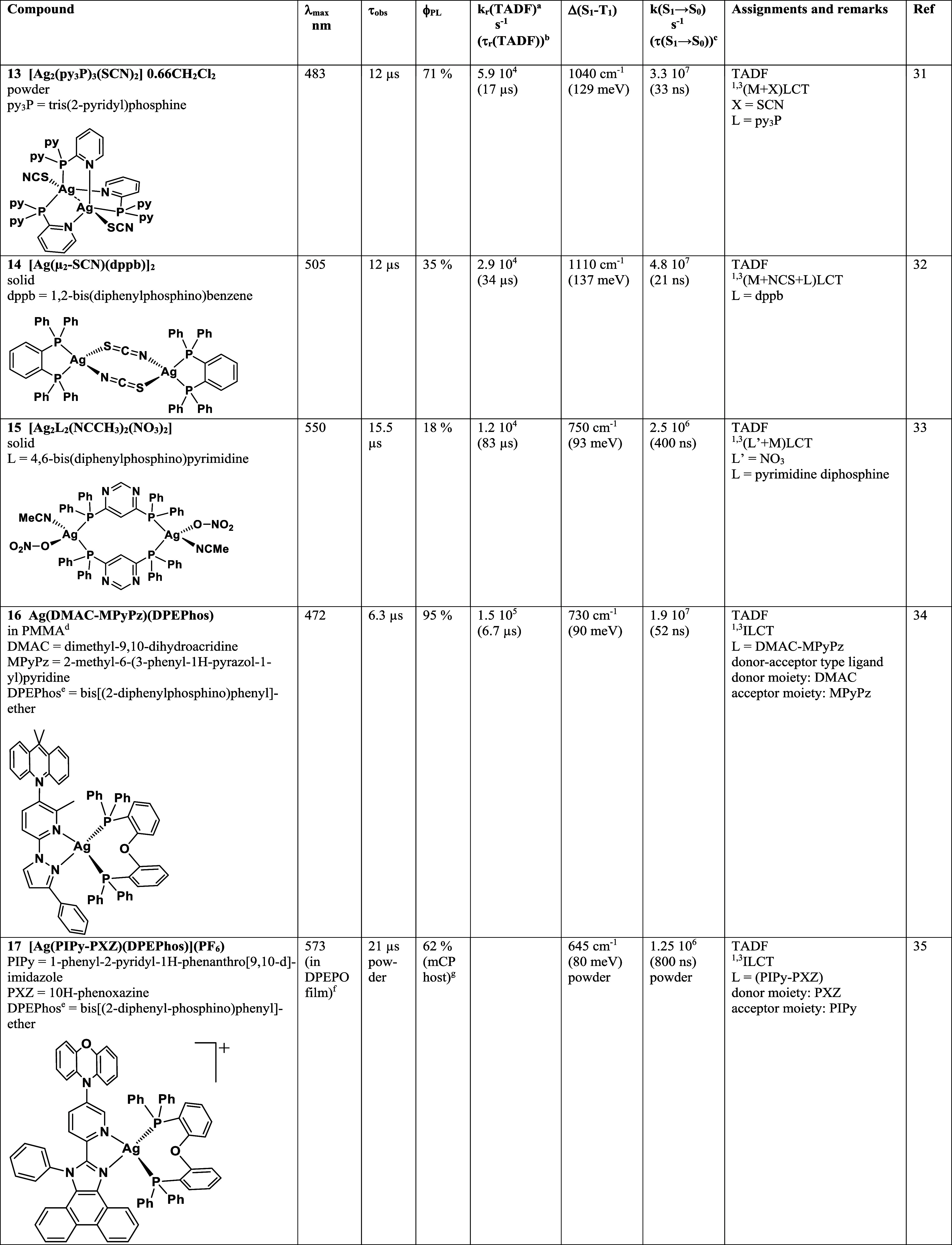

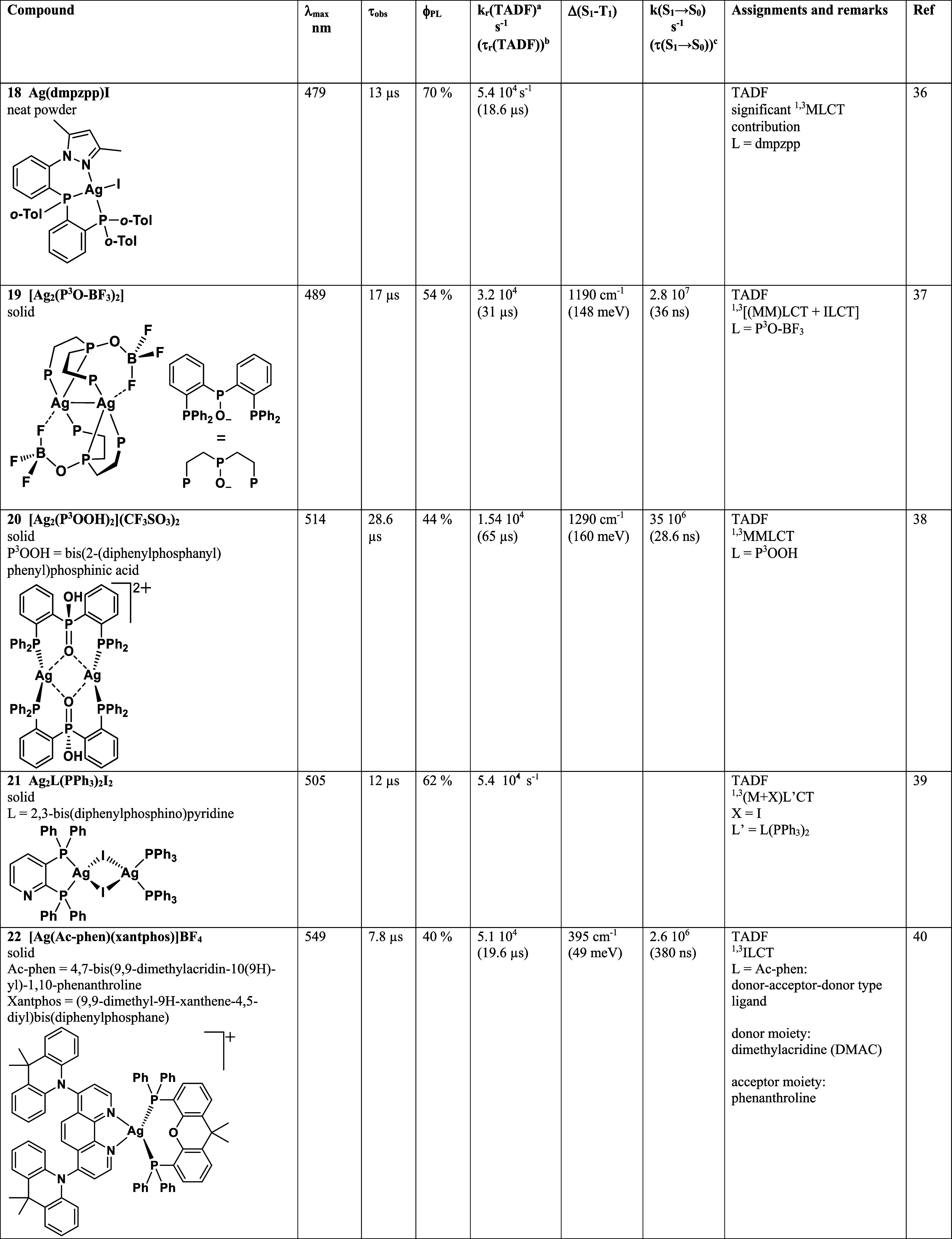

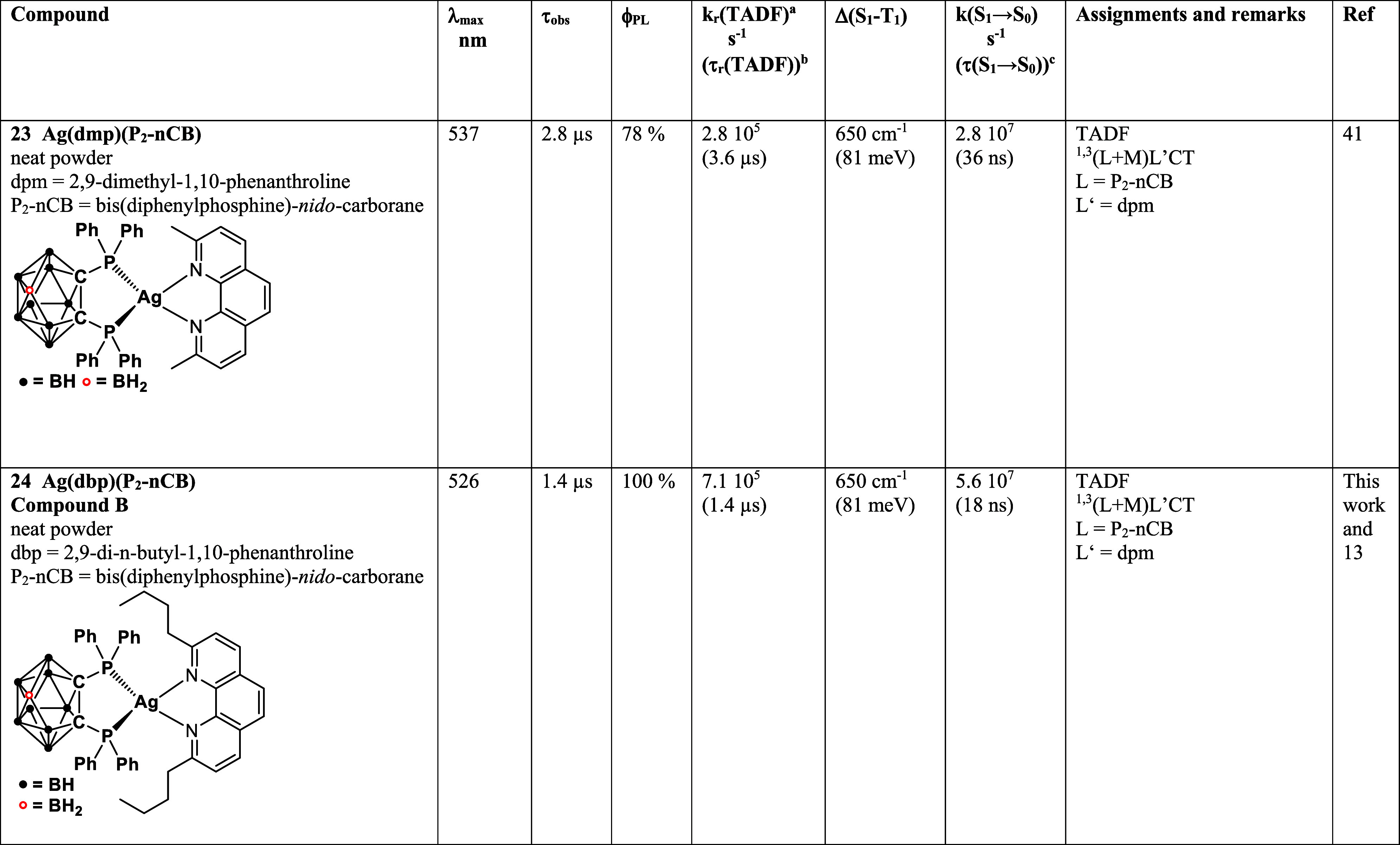
Ag­(I) Complexes Showing Thermally Activated Delayed Fluorescence

a *k*_r_ = Φ_PL_/**τ**.

b τ_r_ = 1/*k*_r_.

c τ­(S_1_–S_0_) = 1/k­(S_1_–S_0_). This value represents the formally calculated fluorescence decay time under the condition of vanishing ISC processes.

d PMMA = poly­(methyl methacrylate).

e DPEPhos = bis­[(2-diphenylphosphino)­phenyl]-ether frequently also abbreviated as POP.

f DPEPO = bis­[2-(diphenylphosphino)­phenyl]­ether oxide.

g mCP = 1,3-bis­(*N*-carbazolyl)­benzene.

Inspection of the compounds listed in Table 2 shows that different TADF design strategies have been employed:

(a) In a first strategy, specific ligands are used that exhibit low-energy intraligand CT states, representing ^1,3^ILCT states. Examples, summarized in Table 2, are the compounds **16**,^34^ **17**,^35^ and **22**.^40^ A very recent publication reports a further example of low-lying ^1,3^ILCT states showing a very high photoluminescence quantum yield of Φ_PL_ = 98%.^42^ In these compounds, the lowest singlet and triplet excited states are localized within the ligand that is coordinated via nitrogen atoms and functionalized with a donor moiety. The singlet–triplet energy gap between these ^1,3^ILCT states is small due to the charge transfer within the ligand. Presumably, for these compounds, the Ag­(I) 4d orbitals are less involved in the ILCT states, thus leading to relatively weak SOC and relatively long ISC time between singlet and triplet states.
(b) In a second design strategy, two different ligands are selected. One of them, ligand L or D, shows pronounced electron-donating properties, while the second one, ligand L′ or A, exhibits electron acceptor character. In suitable situations, the resulting ^1,3^LL’CT states represent the lowest excited ones. As the two involved ligands are spatially well-separated, orbital overlap of the D and A moieties is relatively small. This results in small energy gap. Hence, occurrence of TADF is often observed. Compound **8** ^28^ represents such an example (Table 2). As the states’ wave functions involve both ligands with the metal M in between, frequently, metal d-orbital character mixes to the ^1,3^LL’CT wave functions. Thus, these states are better characterized as ^1,3^(L+M)­L’CT states. Corresponding examples are compounds **12**,^30^ **15**,^33^ **23**,^41^ and **24**.^13^ It is expected that in case of strategy (b), the SOC effect is stronger and that ISC times are significantly shorter than for materials of design strategy (a).
(c) In a third strategy, TADF compounds can also be designed on the basis of low-lying excited states that are dominated by MLCT including halide X character or other anion contribution, leading to ^1,3^(M+X)­LCT states. Examples given in Table 2 are compounds **9**,^10^ **10**,^29^ **11**,^23^ **13**,^31^ **14**,^32^ **21**,^39^ and probably compound **18**.^36^ Moreover, dimers with direct Ag–Ag contact, leading to low-lying states with ^1,3^MMLCT character, such as dimers **19** ^37^ and **20**,^38^ are also reported.

It is noted that the compounds discussed in Tables 1 and 2 were studied in different environments. Mostly, neat powder materials were investigated. This is justified as emission quenching caused by the enhanced external rigidity induced by the crystalline environment is drastically decreased compared to fluid solution^4^ (compare also ref 43). External rigidity might be less important for molecules that already exhibit high internal molecular rigidity.^4,9,44^ In other cases, however, low-concentration doping in host materials, such as PMMA, was applied. This was necessary, as for these compounds, radiationless energy transfer between the chromophores had to be prevented in order to minimize concentration quenching.

Comparison of the compounds summarized in Table 2 shows that compound **24** (= compound **B**) exhibits outstanding TADF properties, in particular, with respect to the emission quantum yield of Φ_PL_ = 100% and ultrashort radiative TADF decay time of only 1.4 μs. These decay properties compare well with those of Ir­(ppy)_3_,^14^ a compound frequently applied as efficient OLED emitter. Detailed photophysical properties of this landmark silver complex are discussed in Sections 5 and 6 on the basis of experimental and theoretical studies, respectively.

## 3 Long-Lived Ambient-Temperature Phosphorescence of [Ag(dmp)(DPEPhos)]^+^ and Large Singlet–Triplet Energy Gap

In this section, we discuss, in particular, the luminescence properties of [Ag­(dmp)­(DPEPhos)]­PF_6_ (compound **A**) shown in Figure 1. At ambient temperature, the neat powder of [Ag­(dmp)­(DPEPhos)]­PF_6_ does not show any detectable emission even under a N_2_ atmosphere. However, when doped in PMMA at low concentration (c ≪ 1 wt %) and under N_2_ purging, both concentration quenching and oxygen quenching are largely suppressed. Thus, bright emission with Φ_PL_ = 50% is found. The decay time observed for this sample (carried out in vacuum at p ≈10^–6^ bar) is extremely long amounting to τ_obs_ = 110 ms. Thus, the radiative decay time is as long as τ_r_ = τ_obs_/Φ_PL_ = 220 ms, corresponding to the very slow radiative rate of *k*_r_ = 4.5 s^–1^. Hence, the emission observed represents a spin-forbidden phosphorescence.^12^

Figure 3 reproduces the ambient-temperature emission and absorption spectra of compound **A**. The emission displays vibrational progression of ≈1400 cm^–1^, which is much better resolved at *T* = 77 K (Figure 4). This spectral structure resembles largely the ligand-centered phosphorescence spectrum of [Rh­(phen)_3_]­(PF_6_) in glycerol at *T* = 77 K. It also shows a ≈1400 cm^–1^ vibrational progression that displays vibrations of the phen (=1,10-phenanthroline) ligand.^45^ As dmp and phen feature the same chromophore core, it can be expected that the same vibrations participate in the emission process. Hence, compound **A** phosphorescence is assigned to T_1_ = ^3^LC­(dmp) → S_0_ transitions. This is in line with the calculations presented in Section 6. Furthermore, there is no significant change in spectral shape with temperature variation, which agrees with the given assignment and is in contrast to the situation usually found for Cu­(I)- or Ag­(I)-based TADF emitters (see below).^4,6^

For ligand-centered excitations, the exchange interaction and thus the related singlet–triplet gap Δ*E*(S_1_–T_1_) are expected to amount to several thousand cm^–1^ (several hundred meV).^15^ Subsequently, we estimate this energy gap from the experimental data. The T_1_ → S_0_ 0–0 transition energy is determined from the high-energy onset of the (moderately) well-resolved 77 K emission spectrum reproduced in Figure 4 to ≈22.45 × 10^3^ cm^–1^ (2.78 eV). The corresponding singlet state transition should be displayed in the absorption spectrum reproduced in Figure 3. At first sight, one might identify the stronger absorptions below ≈300 nm (≈33.3 × 10^3^ cm^–1^, 4.13 eV) with molar extinction coefficients of ε > 10000 M^–1^ cm^–1^ as suitable candidates. This would correspond to an energy gap of at least around 10.9 × 10^3^ cm^–1^ (1.35 eV). However, for the coordinated dmp ligand with an extended ππ* electronic system involving, though only marginally, also other parts of the complex, this gap is expected to be much too large.^15^ Therefore, a better candidate for the S_0_ → S_1_ transition, marked in the absorption spectrum, is the relatively weak peak at 354 nm with a molar extinction coefficient of around 1 × 10^3^ M^–1^ cm^–1^ (Figure 3). Using the 0–0 transition energy corresponding to this absorption peak, estimated to 27.6 × 10^3^ cm^–1^ (3.42 eV), the energy gap can be evaluated to Δ*E*(S_1_–T_1_) = 5.15 × 10^3^ cm^–1^ (0.64 eV) (Figure 5). This value fits much better to the tendency described for singlet–triplet separations in organic molecules with extended ππ* systems.^15^ Indeed, from measured fluorescence and phosphorescence spectra of 1,10-phenanthroline (phen), a gap was determined amounting to Δ*E*(S_1_–T_1_) ≈7.1 × 10^3^ cm^–1^ (0.88 eV).^46,47^ Moreover, for phen coordinated to Zn­(II) in ZnCl_2_(phen), highly resolved fluorescence and phosphorescence spectra measured at *T* = 4.2 K provide a gap of Δ*E*(S_1_–T_1_) ≈5.2 × 10^3^ cm^–1^ (0.65 eV) (Table 3).^48^ This latter value fits well with the gap determined for compound **A**. In conclusion, at this large energy separation, up-ISC can be neglected at ambient temperature.

**Table 3:** Photophysical Data of [Ag­(dmp)­(DPEPhos)]^+^ and Singlet and Triplet State Energies of Zn­(phen)­Cl_2_ and 1,10-Phenanthroline (phen) Exhibiting ^3^LC → S_0_ Transitions

properties	[Ag(dmp)(DPEPhos)]^+^ compound **A** (this work and 12)	Zn(phen)Cl_2_ ^a^ ref 48	phen^b^ refs 46 and 47
**T**_**1**_	22.45 × 10^3^ cm^–1^ (2.78 eV)^c^	22.1 × 10^3^ cm^–1^ (2.74 eV)	22.7 × 10^3^ cm^–1^ (2.82 eV)
**S**_**1**_	27.6 × 10^3^ cm^–1^ (3.42 eV)^d^	27.3 × 10^3^ cm^–1^ (3.39 eV)	29.8 × 10^3^ cm^–1^ (3.7 eV)
**Δ*E*** (**S**_**1**_–**T**_**1**_)	5.15 × 10^3^ cm^–1^ (0.64 eV)	5.2 × 10^3^ cm^–1^ (0.65 eV)	7.1 × 10^3^ cm^–1^ (0.88 eV)
**Φ**_**PL**_(**air**)^e^	2%		
**Φ**_**PL**_(**N**_**2**_)^f^	50%		
**τ**_**obs**_ ^g^	110 ms		
τ_**r**_ = **τ**_**obs**_/**Φ**_**PL**_ ^h^	220 ms		
***k***_**r**_	4.5 s^–1^		
**ISC time** ^i^	9 ps		

a Data from highly resolved 0–0 transitions measured from neat crystals at *T* = 4.2 K.

b Estimated 0–0 transitions from fluorescence and 77 K phosphorescence spectra of 1,10-phenanthroline dissolved in CH_2_Cl_2_.

c 0–0 transition energy estimated from the phosphorescence spectrum shown in Figure 4.

d 0–0 transition energy estimated from the absorption peak at 354 nm marked in Figure 3.

e Emission quantum yield of compound **A** doped in PMMA at c ≪ 1 wt % under ambient conditions.

f Emission quantum yield of compound **A** doped in PMMA at c ≪1 wt %, 300 K, N_2_ purged.

g Compound **A** doped in PMMA at c ≪1 wt % measured at 300 K in vacuum at *p* < 10^–6^ bar.

h Radiative decay time.

i Calculated, see Section 6.

Quantum mechanical perturbation theory allows us to get qualitative insight into understanding the very slow T_1_ → S_0_ transition rate. This rate can be expressed by (deduced from ref 49):

$$k_{r}\left(T_{1}\left(j\right)-S_{0}\right)=64\pi^{4}\nu^{3}{\left(3{hc}^{3}\right)}^{-1}\left\{|\left\langle T_{1}\left(j\right)|H_{\text{SO}}|S_{1}\right\rangle /\Delta E\left(S_{1}-T_{1}\right){|}^{2}\right\}\cdot \left\{|\left\langle S_{0}|e\cdot r|S_{1}\right\rangle {|}^{2}\right\}$$ 1

For visualization, we assume in a simplifying model that the energetically nearest S_1_ state represents the dominant mixing state of singlet character and that it essentially interacts via SOC with only one triplet substate *T*_1_(*j*). ν is the emission frequency, and c is the velocity of light. The coefficient {|**⟨** *T*_1_(*j*)|H_SO_|S_1_ **⟩ /** Δ*E*(S_1_–T_1_)|^**2**^} displays the extent of mixing of singlet character to the triplet substate *T*_1_(*j*) given by the squared SOC matrix element (SOCME) divided by the squared energy gap between the triplet and the admixing singlet S_1_. For obtaining fast radiative rate *k*_r_, (i) the perturbing state S_1_ should be proximate in energy to the T_1_ state and (ii) the squared transition dipole matrix element {|**⟨**S_0_|e·**r**|S_1_ **⟩|** ^**2**^} that provides intensity to the initially spin-forbidden triplet–singlet transition should be large (strongly allowed transition). However, for [Ag­(dmp)­(DPEPhos)]^+^, the energy gap in the denominator is relatively large amounting to Δ*E*(S_1_–T_1_) = 0.64 eV (Table 3), the singlet S_0_↔S_1_ transition strength is small (weak absorption at 354 nm, Figure 3, and small calculated oscillator strength, Table 7 below), and the SOCME is very small (0.21 cm^–1^, 0.03 meV, Table 7 below). Accordingly, the very slow *k*_r_(T_1_) rate of 4.5 s^–1^, as experimentally determined, is well-understandable.

**Table 4:** Emission Data of Ag­(dbp)­(P_2_-nCB) Neat Powder (Compound **B**) and Dissolved in PMMA at ≈1 wt %^a^

properties	powder	PMMA
λ_max_ (300 K)	526 nm	535
Φ_PL_ (300 K)	100%	85%
τ (300 K)	1.4 μs	
*k*_r_ (300 K)	7.1 × 10^5^ s^–1^	
Φ_PL_ (77 K)	87%	
τ (77 K)	1300 μs	
*k*_r_ (77 K)	6.7 × 10^2^ s^–1^	
τ_r_ (77 K)	1490 μs	
*k*_nr_ (77 K)	1 × 10^2^ s^–1^	
τ (T_1_, 40 K) (plateau)	1570 μs	
*k*_r_(plateau)	5.5 × 10^2^ s^–1^	
*k*_r_(S_1_ → S_0_)^b^	5.6 × 10^7^ s^–1^ (18 ns)	
E(0–0, T_1_ → S_0_)^c^	≈20340 cm^–1^	
Δ*E*(S_1_–T_1_)^b^	650 cm^–1^ (80 meV)	
ISC time^d^	20 ps	

a Adapted from ref 13.

b Determined from the fit of eq 2 to the experimental luminescence decay times measured for a powder sample of Ag­(dbp)­(P_2_-nCB) at different temperatures.

c The 0–0 transition energy is estimated from the blue-side flank of the 40 K emission spectrum (Figure 7), taking the crossing point of the tangent line with the zero-emission line.

d Calculated, see Section 6.

**Table 5:** Energies of the Low-Lying Singlet and Triplet States and Corresponding Molecular Orbital (MO) Transitions Calculated for [Ag­(dmp)­(DPEPhos)]^+^ **A** and Ag­(dbp)­(P_2_-nCB) **B** at the M06-2X/def2-TZVP Level Using the Optimized T_1_ State Geometry

state	E, eV	contributions/%
		**[Ag**(**dmp**)(**DPEPhos**)**]** ^+^ **A**
S_1_	3.84	HOMO–1 → LUMO (65%), HOMO → LUMO (29%)
T_1_	2.32	HOMO–1 → LUMO (72%), HOMO → LUMO (22%)
T_2_	3.25	HOMO–1 → LUMO+1 (65%), HOMO → LUMO+1 (25%), HOMO–1 → LUMO (3%)
S_2_	3.92	HOMO–1 → LUMO+1 (52%), HOMO → LUMO+1 (27%), HOMO–11 → LUMO (8%)
		**Ag**(**dbp**)(**P**_**2**_-**nCB**) **B**
S_1_	2.48	HOMO → LUMO (92%), HOMO–1 → LUMO (3.4%)
T_1_	2.31	HOMO → LUMO (90%), HOMO–1 → LUMO (3.4%)

**Table 6:** Orbital Energies and Molecular Fragment Contributions from Mulliken Population Analysis Calculated for [Ag­(dmp)­(DPEPhos)]^+^ **A** and Ag­(dbp)­(P_2_-nCB) **B**, at the M06-2X/def2-TZVP Level Using the Optimized T_1_ State Geometry

[Ag(dmp)(DPEPhos)]^+^ A					Ag(dbp)(P_2_-nCB) B				
orbital	energy, eV	contribution, %			orbital	energy, eV	contribution, %		
		dmp	Ag	DPEPhos			dbp	Ag	P_2_-nCB
LUMO+1	–3.13	96	2	2	LUMO+1	–1.42	100		
LUMO	–3.66	99			LUMO	–1.89	97	1	2
HOMO	–9.18	20	12	68	HOMO	–6.02	4	16	80
HOMO–1	–9.42	80	2	18	HOMO–1	–6.41		2	98

**Table 7:** TD-DFT-Calculated (M06-2X/def2-TZVP) Energies of Singlet State S_1_ and Lower-Lying Triplet States T_n_ (in eV), Spin–Orbit Coupling Matrix Elements **⟨**S_1_|H_SO_|T_1_ **⟩** (in cm^–1^) and Corresponding ISC Rate Constants k­(ISC) (in s^–1^), and the Radiative Decay Times (in ms) of the T_1_ States of Compounds **A** and **B** in the Ground State S_0_ and the Optimized S_1_ and T_1_ Geometries

	[Ag(dmp)(DPEPhos)]^+^ A					Ag(dbp)(P_2_-nCB) B				
relaxed geometry	S_0_		S_1_	T_1_		S_0_		S_1_	T_1_	
**state energies**										
properties	exp	calc	calc	exp	calc	exp	calc	calc	exp	calc
S_1_, eV	3.42^a^	4.24	3.03		3.84	2.82^b^	3.46	2.63	2.60^c^	2.48
*f*(S_0_ → S_1_)^d^		0.016					0.052			
*f*(S_1_ → S_0_)^e^			0.027					0.071		
T_1_, eV		3.34	2.88	2.78^a^	2.32		3.28	2.53	2.52^f^	2.31
Δ*E*(S_1_–T_1_)				0.64^a^	1.52				0.08^f^	0.17
T_2_, eV		3.65	–		3.25		3.37	–		–
T_3_, eV		3.92	–		–		–	–		–
T_4_, eV		4.03	–		–		–	–		–
T_5_, eV		4.09	–		–		–	–		–
**SOCMEs**, **ISC rates**, **and radiative decay times**										
**⟨**S_1_|H_SO_|T_1_ **⟩**			14.79		0.21			8.85		12.46
k(ISC)(S_1_ → T_1_)			1.1 × 10^11^ (9 ps)					4.95 × 10^10^ (20 ps)		
τ_r_(T_1_ → S_0_)				220^a^	1660				1.57^f^	2.71

a From Table 3.

b Estimated from the low energy flank of the absorption band with its peak maximum at 385 nm as displayed in Figure 7.

c E­(0–0) + Δ*E*(S_1_–T_1_), both values are taken from Table 4.

d Oscillator strength of the S_0_ → S_1_ transition.

e Oscillator strength of the S_1_ → S_0_ transitions.

f From Table 4.

Emission quenching occurring in neat [Ag­(dmp)­(DPEPhos)]­PF_6_ is addressed briefly again. Upon excitation, ligand-centered excited states exhibit only moderate geometry changes compared to the electronic ground state, as expressed by occurrence of resolved vibrational structures in low-temperature emission spectra. As a consequence, the resonance condition for efficient energy transfer between neighboring molecules in neat material is fulfilled. Thus, excitation energy transfer and subsequent quenching, for example, by triplet–triplet annihilation or always existing impurities, are very probable for long-lived excited states, such as the T_1_ state of the discussed material **A**. Hence, efficient concentration quenching can occur. Due to these processes, emission detection requires low emitter concentration, for example, doping in PMMA. For completeness, it is noted that frequently, concentration quenching is not relevant for Cu­(I) and Ag­(I) TADF complexes due to the self-trapping mechanism associated with significant geometry reorganization^50^ in the emitting state. As a consequence, resonance energy transfer is largely prevented in these TADF materials (see Section 5 and refs 4, 6, 8, and 13 ).

Another efficient quenching process is known to be induced by oxygen. Therefore, even at very dilute concentration of the triplet emitter molecule, for example, at c ≪ 1 wt % doped in PMMA, only quantum yields of Φ_PL_(air) = 2% are found under ambient conditions. But under N_2_ purging, Φ_PL_(N_2_) increases to 50% at ambient temperature. According to the extremely long emission decay time of 110 μs and the high emission quantum yield at ambient temperature, it is suggested to apply the outstanding material [Ag­(dmp)­(DPEPhos)]^+^ as oxygen sensor material, in particular, as a very wide range of oxygen concentrations can be displayed via the oxygen-dependent emission decay time (compare ref 51). Table 3 and Figure 5 summarize the photophysical data determined for [Ag­(dmp)­(DPEPhos)]­PF_6_ (compound **A**) and related compounds.

## 4 Chemical Tuning: From Ligand-Centered to Ligand-to-Ligand Charge Transfer States

As discussed in Section 3, the lowest excited state of [Ag­(dmp)­(DPEPhos)]^+^ (**A**) shows ^3^LC­(ππ*) character localized at the dmp ligand with relatively large energy separation to the next higher-lying singlet state (Figure 6 left). Moreover, 4d­(Ag) orbitals are only marginally involved, as they lie energetically relatively deep. Thus, SOC is weak, resulting in long-lived phosphorescence. This is attractive, for example, for oxygen sensor materials but not for OLED applications. For these latter, the design of compounds exhibiting fast TADF is desired. Such a change can be accomplished by involving the second ligand in the formation of the lowest excited states, for example, by modifying the DPEPhos ligand to become distinctly electron donating, while the dmp ligand may largely be kept unchanged. In particular, this is achieved, if orbitals of the modified ligand are energetically destabilized (Figure 6 right). As a consequence, a donor–acceptor situation is obtained with L2, representing the donor, and L1 the acceptor, giving low-lying ^1,3^L2L1CT = ^1,3^LL’CT states. As in this case, the two unpaired electrons involved largely reside on spatially separated ligands; it is expected that the exchange interaction between these two particles and hence the energy gap Δ*E*(S_1_–T_1_) are drastically reduced compared to the gap occurring for ^1,3^LC states. Moreover, it is expected that some 4d­(Ag) contribution is involved in the LL’CT states. Thus, it is indicated that the resulting lowest excited states have to be characterized as ^1,3^(L + M)­L’CT states. The importance of the 4d­(Ag) contribution will be displayed in the size of the SOC matrix element or the radiative phosphorescence decay time (compare eq 1). Indeed, this is observed, as discussed in Sections 5 and 6.

**Figure 6:**
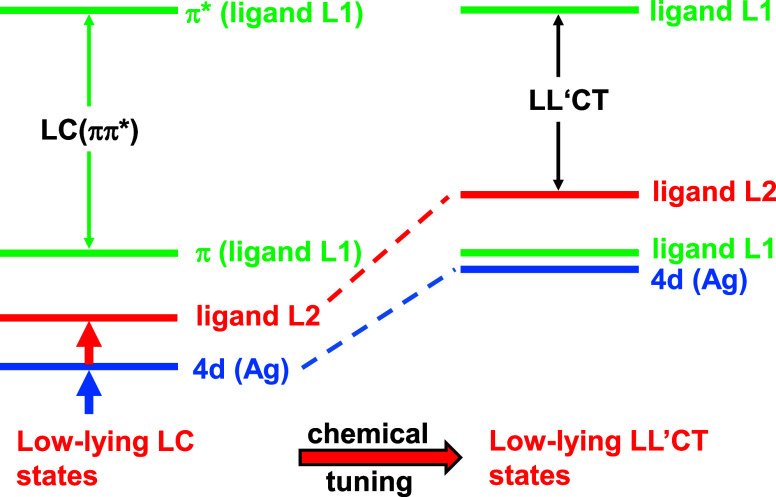
Schematic and simplified diagram to illustrate a material design concept for modifying a compound with low-lying ligand-entered (LC) energy states to a compound exhibiting low-energy ligand-to-ligand charge transfer (LL’CT) states by chemical tuning. The transitions occurring within ligand L1 are usually termed LC transitions. The red and blue arrows shown in the left diagram represent the effects required for orbital energy shifts. Such a procedure, introducing an electron-donating ligand, allows us to modify a phosphorescent complex to become a TADF emitter. The charge transfer transitions from ligand L2 to ligand L1 are usually labeled as LL’CT transitions. Frequently, additionally, metal d-character contributes to these states. This leads to ^1,3^(L+M)­L’CT states. For completeness, it is noted that for a detailed discussion with respect to specific compounds, additional orbitals may be involved. This is discussed in Section 6.

In fact, the design concept described can be realized by replacing DPEPhos with P_2_-nCB (compare Figures 1 and 2). Formally, both compounds are similar, as “only” the phenyl ether group is replaced by the negatively charged *nido*-carborane cage, while the phenanthroline substitutions, dimethyl and dibutyl, are electronically rather similar. However, introduction of the negatively charged *nido*-carborane cage has a crucial impact due to its pronounced electron-donating property. Thus, this modified complex, compound **B**, represents a TADF emitter with a relatively small singlet–triplet gap. Indeed, this is demonstrated in the next section and in ref 13. For completeness, it is mentioned that the concept described is basically also realized designing the compounds **8**,^28^ **12**,^30^ **15**,^33^ and **23**.^41^

## 5 Ultrashort-Lived TADF of Ag(dbp)(P_2_-nCB)

First insight into the photophysical properties of Ag­(dbp)­(P_2_-nCB), compound **B** (Figure 2), can already be deduced from simple steady-state spectral studies. The absorption spectrum, reproduced in Figure 7, shows intense bands below 350 nm. These are ascribed to ππ* transitions within the dbp and the P_2_-nCB ligands, while the less intense and broad absorption band centered at 385 nm is assigned to transition(s) with pronounced ligand L­(P_2_-nCB)-to-L′(dbp) charge transfer character (compare Section 6). The emission spectra, also displayed in Figure 7, are broad with a half-width of ≈3300 cm^–1^ (400 meV) at ambient temperature and cannot be better resolved even when cooled to *T* = 40 K. Such spectral features are typical for CT transitions,^4,6,8,13^ as these couple strongly to molecular vibrations and local phonons that result in pronounced spectral broadening. The relatively small blueshift observed with temperature increase from 40 to 300 K represents an indication of occurrence of TADF, as the low temperature emission refers to the T_1_ → S_0_ phosphorescence, while the emission at 300 K, induced by thermal population, is dominated by delayed S_1_ → S_0_ fluorescence (TADF). The corresponding energy shift may coarsely be estimated from the blue-side flanks of the emission spectra by the crossing energy of the tangent line with the zero-emission line. This gives ≈900 cm^–1^ (110 meV). Alternatively, one can determine the energy separation of the emission maxima, amounting to ≈500 cm^–1^ (62 meV). In conclusion, the spectral blue shift occurring with temperature increase approximately reflects the energy gap Δ*E*(S_1_–T_1_). Later in this section, a more accurate method is presented.

**Figure 7:**
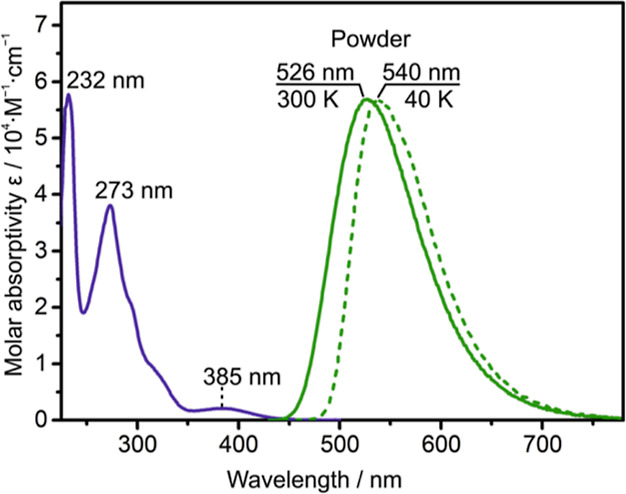
Absorption and emission spectra of Ag­(dbp)­(P_2_-nCB), compound **B**. Absorption: The compound was dissolved in dichloromethane at a concentration of ≈10^–5^ M and measured at *T* = 300 K. Photoluminescence: The neat powder was excited at 410 nm, and the emission was measured at 300 and 40 K, respectively. Adapted with permission from ref 13. Copyright 2017, American Chemical Society.

Very frequently, Cu­(I) and Ag­(I) TADF complexes exhibit a high emission quantum yield. In particular, the quantum yield of Ag­(dbp)­(P_2_-nCB) powder is as high as Φ_PL_ = 100% at ambient temperature. This is a consequence of three effects: (i) The CT transition induces geometry distortions relative to the electronic ground state that stabilize the excited states’ energies.^50^ Accordingly, the resonance condition for fast nonradiative energy transfer to nonexcited neighbor molecules is no longer fulfilled. Hence, the excitation energy is trapped in the originally excited molecule. Thus, concentration quenching is negligible.^4,8^ (ii) The fast radiative S_1_ → S_0_ rate of *k*_r_(S_1_–S_0_) = 5.6 × 10^7^ s^–1^ (corresponding to a radiative fluorescence decay time of 18 ns, see below in this section) strongly predominates nonradiative deactivation to the ground state S_0_. (iii) Rigidity of the neat material of compound **B** restricts too pronounced geometry changes that normally are responsible for nonradiative quenching. Two factors control the rigidity: the internal molecular rigidity (essentially given by the sterically very demanding 2,9-butyl substitutions at the phenanthroline ligand) and the external rigidity (caused by the rigid crystalline environment).^4,44^

Interestingly, for the neat compound **B**, concentration quenching is even insignificant at drastic increase of the emission decay time by 3 orders of magnitude from τ_r_ = 1.4 μs at *T* = 300 K to τ_r_ = 1490 μs observed at *T* = 77 K. In this case, the emission quantum yield is only slightly diminished to Φ_PL_ = 87% (Table 4 below).^13^ This slight reduction probably results from nonradiative molecular processes that become competitive to the radiative ones.

In contrast, the reduction of rigidity has an influence on the emission quantum yield. For example, upon lowering the internal molecular rigidity, as realized in compound **23** (Ag­(dmp)­(P_2_-nCB) (Table 2) that carries sterically less demanding methyl substitutions at the phen ligand, Φ_PL_ drops to 78%.^41^ Similarly, upon diminishing the external rigidity by doping Ag­(dbp)­(P_2_-nCB), **B**, in a relatively soft PMMA matrix, Φ_PL_ is reduced to 85% (Table 4 below).^13^

Obviously, for Ag­(dbp)­(P_2_-nCB) neat powder exhibiting a quantum yield of Φ_PL_ = 100%, the balance between necessary geometry change in the excited state for prevention of concentration quenching and rigidity of the emitter molecule for reduction of nonradiative quenching has just found an optimum. A related study shows that extreme molecular rigidity leads even to lower quantum yield in neat powder material as concentration quenching then becomes possible.^4,44^

For a more detailed experimentally based classification of TADF properties, steady-state spectral studies do not seem to provide essential information. Therefore, we investigated the temperature dependence of the emission decay dynamics. In a three-state model, consisting of the electronic ground state S_0_, the excited S_1_ state, and the (triply degenerate) T_1_ state, the temperature dependence of the molecule’s decay time τ­(T) can be expressed by a modified Boltzmann equation if fast equilibration between the two excited states is established. In this situation, monoexponential decay is given.^4,13,52,53^

2 $$\tau \left(T\right)=\frac{3+\exp\left[-\frac{\Delta E\left(S_{1}-T_{1}\right)}{k_{B}T}\right]}{3k\left(T_{1}\right)+k\left(S_{1}\right)\exp\left[-\frac{\Delta E\left(S_{1}-T_{1}\right)}{k_{B}T}\right]}$$

τ­(*T*) represents the emission decay time at a given temperature *T*, *k*(T_1_) and k­(S_1_) are the rate constants for the transitions from the respective excited states to the electronic ground state, Δ*E*(S_1_–T_1_) is the energy gap between the S_1_ and T_1_ states, and k_B_ is the Boltzmann constant.

In Figure 8 left, we reproduce emission decay curves measured at selected temperatures, and in Figure 8 right, the corresponding decay constants are plotted versus temperature.

**Figure 8:**
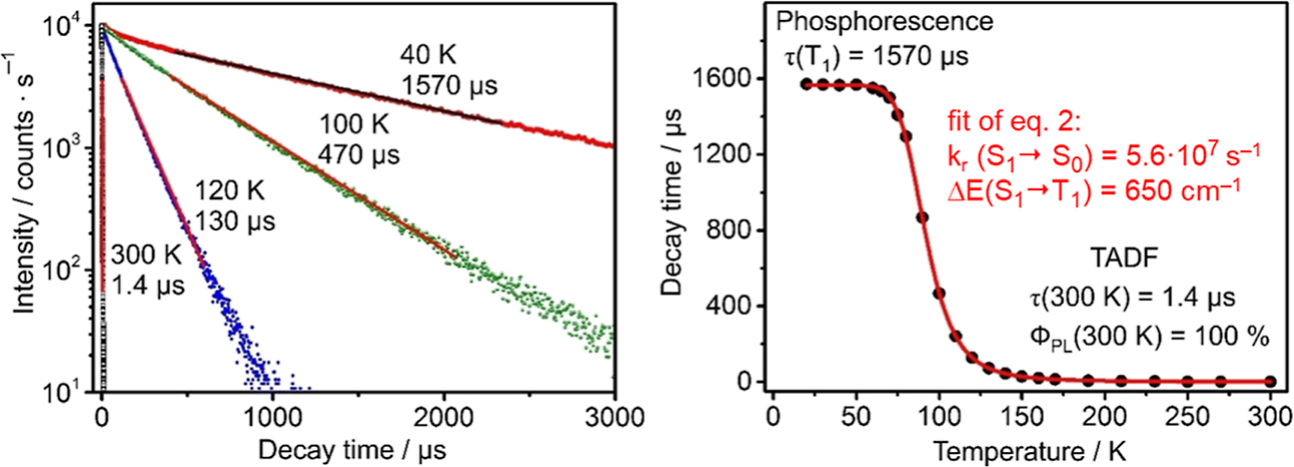
Left: Luminescence decay curves and times of neat Ag­(dbp)­(P_2_-nCB) powder (compound **B**) were measured at selected temperatures. Right: Emission decay times plotted versus temperature. The values of *k*_r_(S_1_ → S_0_) = 5.6 × 10^7^ s^–1^ (18 ns) and ΔE­(S_1_–T_1_) = 650 cm^–1^ (80 meV) result from a fit of eq 2 to the experimental τ­(T) plot, fixing τ­(T < 60 K) to 1570 μs as determined for the plateau value. Reproduced with permission from ref 13. Copyright 2017, American Chemical Society.

At low temperature (T < 60 K), a plateau showing a decay time of 1570 μs is observed. This decay corresponds to the T_1_ → S_0_ phosphorescence of the Ag­(dbp)­(P_2_-nCB) (**B**) neat powder. Assuming quantum efficiency Φ_PL_ similar to the one determined at *T* = 77 K of Φ_PL_(77 K) = 87%,^13^ a radiative rate of *k*_r_ = 5.5 × 10^2^ s^–1^ is found for the range of the plateau. This rate corresponds to relatively slow phosphorescence decay being typical for spin-forbidden T_1_ → S_0_ transitions of Cu­(I) and Ag­(I) complexes.^4,6^ In comparison, the value determined for [Ag­(dmp)­(DPEPhos)]^+^ (**A**) is significantly slower, amounting to only *k*_r_ = 4.5 s^–1^ (Table 3), being a factor of ≈120 slower than determined for Ag­(dbp)­(P_2_-nCB). Accordingly, SOC is distinctly more efficient in compound **B** than in compound **A**. A corresponding tendency is also found theoretically, as presented in Section 6.

With a temperature increase above T ≈60 K, the emission decay time of compound **B** decreases. Already at 77 K, a substantial population of the S_1_ state is occurring, and thus, the TADF decay path shortens the emission decay time from τ­(40 K) = 1570 μs to τ­(77 K) = 1300 μs. With further temperature increase, drastic decay reduction occurs, reaching 1.4 μs at *T* = 300 K. This behavior is typical for the TADF effect: With an increasing thermal population of the S_1_ state, the additional fluorescence decay path becomes more and more important, while the role of the phosphorescence is distinctly diminished (compare refs 4 and 54 for an analysis of the interplay between phosphorescence and delayed fluorescence with temperature change). As the corresponding S_1_ → S_0_ fluorescence rate predominates the T_1_ → S_0_ rate by 5 orders of magnitude (*k*_r_(S_1_) = 5.6 × 10^7^ s^–1^ compared to *k*_r_(T_1_) = 5.5 × 10^2^ s^–1^, Table 4), thermally activated fluorescence dominates strongly at ambient temperature.

For completeness, it is mentioned that prompt fluorescence is not detected, at least at the time resolution applied. However, for TADF Cu­(I) complexes, prompt fluorescence occurring in the picosecond time range has been reported. The corresponding fast decay process has been assigned to be dominated by S_1_ → T_1_ ISC processes.^55^ Calculations for Ag­(dbp)­(P_2_-nCB) predict an ISC time of 20 ps (Section 6). These fast ISC (and RISC) processes guarantee fast equilibration between the two lowest excited states.

The fitting procedure as shown in Figure 8 right, using eq 2, provides two important parameters for compound **B**, the energy gap Δ*E*(S_1_–T_1_) = 650 cm^–1^ (80 meV) and the radiative rate of the S_1_ → S_0_ transition, amounting to *k*_r_(S_1_–S_0_) = 5.6 × 10^7^ s^–1^ (formally 18 ns, Figure 9 and Table 4). This rate is significantly faster than that observed for any other tetrahedrally coordinated Cu­(I) or Ag­(I) TADF compound reported so far. It is even by a factor of 5 faster than the S_1_ → S_0_ rate found for a Cu­(I) dimer that was specifically designed to attain fast *k*_r_(S_1_–S_0_) rates and short TADF decay times. The corresponding design strategy was based on a Davydov type^56,57^ electronic coupling between two symmetry-related molecular moieties.^4,58^

**Figure 9:**
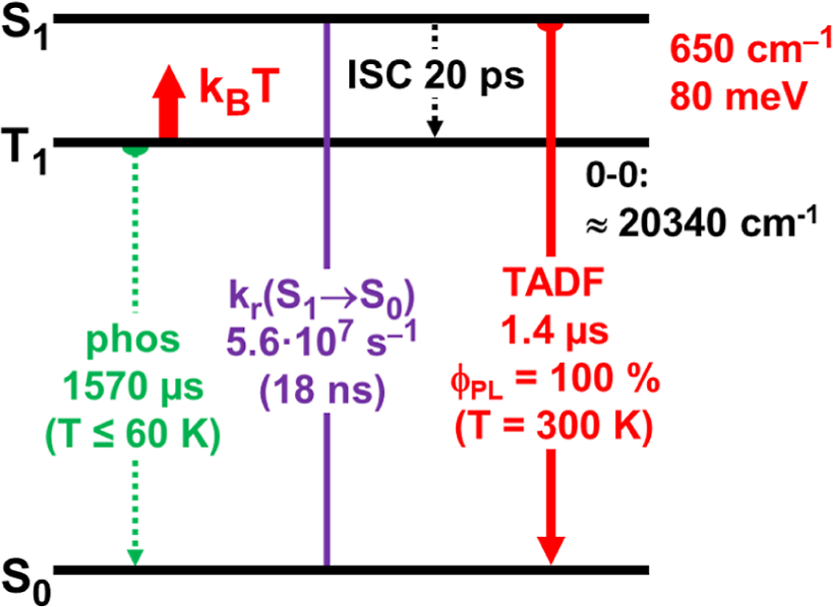
Simplified energy level scheme and decay data for Ag­(dbp)­(P_2_-nCB), compound **B**, neat powder. The ISC time results from calculations as presented in Section 6 (compare ref 13).

In conclusion, the extremely fast *k*_r_(S_1_–S_0_) rate found for Ag­(dbp)­(P_2_-nCB) is a key factor that induces the shortest radiative TADF decay time of 1.4 μs for tetrahedrally coordinated Ag­(I) complexes reported so far. In particular, together with the emission quantum yield of Φ_PL_ = 100%, Ag­(dbp)­(P_2_-nCB) represents a breakthrough TADF material. Potentially, it can be used as an efficient OLED emitter material.

Figure 9 and Table 4 summarize the essential photophysical properties that characterize the outstanding Ag­(dbp)­(P_2_-nCB) TADF material.

## 6 Photophysical Properties of [Ag(dmp)(DPEPhos)]^+^ and Ag(dbp)(P_2_-nCB) Studied by Theoretical Methods

In previous sections, we investigated the photophysical properties of the two complexes [Ag­(dmp)­(DPEPhos)]^+^, **A**, and Ag­(dbp)­(P_2_-nCB), **B**, by experimental methods. In this section, we will present a theoretical DFT and TD-DFT approach to further characterize the low-lying excited states and relaxation dynamics. The results are compared with the experimental ones that are available. Thus, deeper understanding of the compounds’ properties becomes possible.

### 6.1 Theoretical Methods

Calculations were carried out for gas-phase conditions using Density Functional Theory (DFT)^59^ and Time-Dependent DFT (TD-DFT) approaches at the hybrid functional M06-2X^60^/def2-TZVP^61^ level as implemented in the Gaussian16 ^62^ package, for molecular geometry optimizations and excited states calculations, respectively. It is noted that the M06-2X functional has successfully been applied in several related studies.^41,63^ Molecular geometries of compounds [Ag­(dmp)­(DPEPhos)]^+^ and Ag­(dbp)­(P_2_-nCB) were optimized for the electronic ground state S_0_ as well as for the excited singlet S_1_ and triplet T_1_ states. For calculation of the S_1_ → T_1_ intersystem crossing (ISC) rate constants k­(ISC) for the transitions from the S_1_ to T_1_ state, the first five singlet and triplet excited states and corresponding spin–orbit coupling matrix elements (SOCMEs) **⟨**S_1_|H_SO_|T_1_ **⟩** were calculated using optimized S_1_ state geometries with TD-DFT by use of the Gaussian16 and MOLSOC^64^ programs. The intersystem crossing rate constants were computed using the method described in ref 65. Radiative phosphorescence decay times of the T_1_ states τ_r_(T_1_ → S_0_) were calculated using optimized T_1_ state geometries at the TDDFT/CAM-B3LYP^66^/def2-TZVP level of quadratic response theory implemented in the Dalton package.^67^

### 6.2 Theoretical Results and Comparison to Experimental Trends

In the scope of this investigation, we are mainly interested in luminescence and related properties, such as phosphorescence, TADF, and ISC. As the populations of the triplet state T_1_ and the singlet state S_1_, respectively, are crucially involved, it is required to carry out the calculations for the optimized (relaxed) T_1_ and S_1_ state geometries. This is of particular importance as the compounds’ properties investigated in the excited states differ distinctly from those studied, for example, in the electronic ground state S_0_. Table 5 summarizes the energies of low-lying states and the corresponding molecular orbital contributions for the optimized T_1_ state geometry. Obviously, the states are composed of different orbital transitions. This is valid for both compounds. The calculated vertical transition energies S_1_ → S_0_ and T_1_ → S_0_ allow us to roughly estimate the Δ*E*(S_1_–T_1_) energy gaps. Thus, for compound **A**, we obtain Δ*E*(S_1_–T_1_) = 1.52 eV (12260 cm^–1^) and for compound **B**, Δ*E*(S_1_–T_1_) = 0.17 eV (1370 cm^–1^). A pronounced decrease in the gap from **A** to **B** is expected due to the specific design of the complexes. Although this tendency is well-described by the theoretical approach, the thus estimated values are by a factor of roughly two larger than the experimentally determined gaps. These latter one’s amount to 0.64 eV (5150 cm^–1^) for compound **A** (Table 3) and to 80 meV (650 cm^–1^) for compound **B** (Table 4). For completeness, it is noted, referring to compound **B**, that the energy gap calculated from the difference of the vertical transition energies is not expected to match perfectly with the gap determined as the thermal activation energy.

The diagrams of the involved frontier orbitals are displayed in Figure 10. From inspection of both Table 5 and Figure 10, it is seen that singlet S_1_ and triplet T_1_ of compound **A** are similarly and dominantly composed of HOMO–1 → LUMO (S_1_: 65%/T_1_: 72%) transitions and with significant HOMO → LUMO (S_1_: 29%/T_1_: 22%) contributions. The T_2_ state, lying energetically between T_1_ and S_1_, and the S_2_ state, lying close to the S_1_ state, show noticeably different orbital contributions (Table 5). For compound **B**, the S_1_ and T_1_ states are almost equally composed, though in this case, the HOMO → LUMO contribution (S_1_: 92%/T_1_: 90%) distinctly predominates the HOMO–1 → LUMO (3.4%) one.

**Figure 10:**
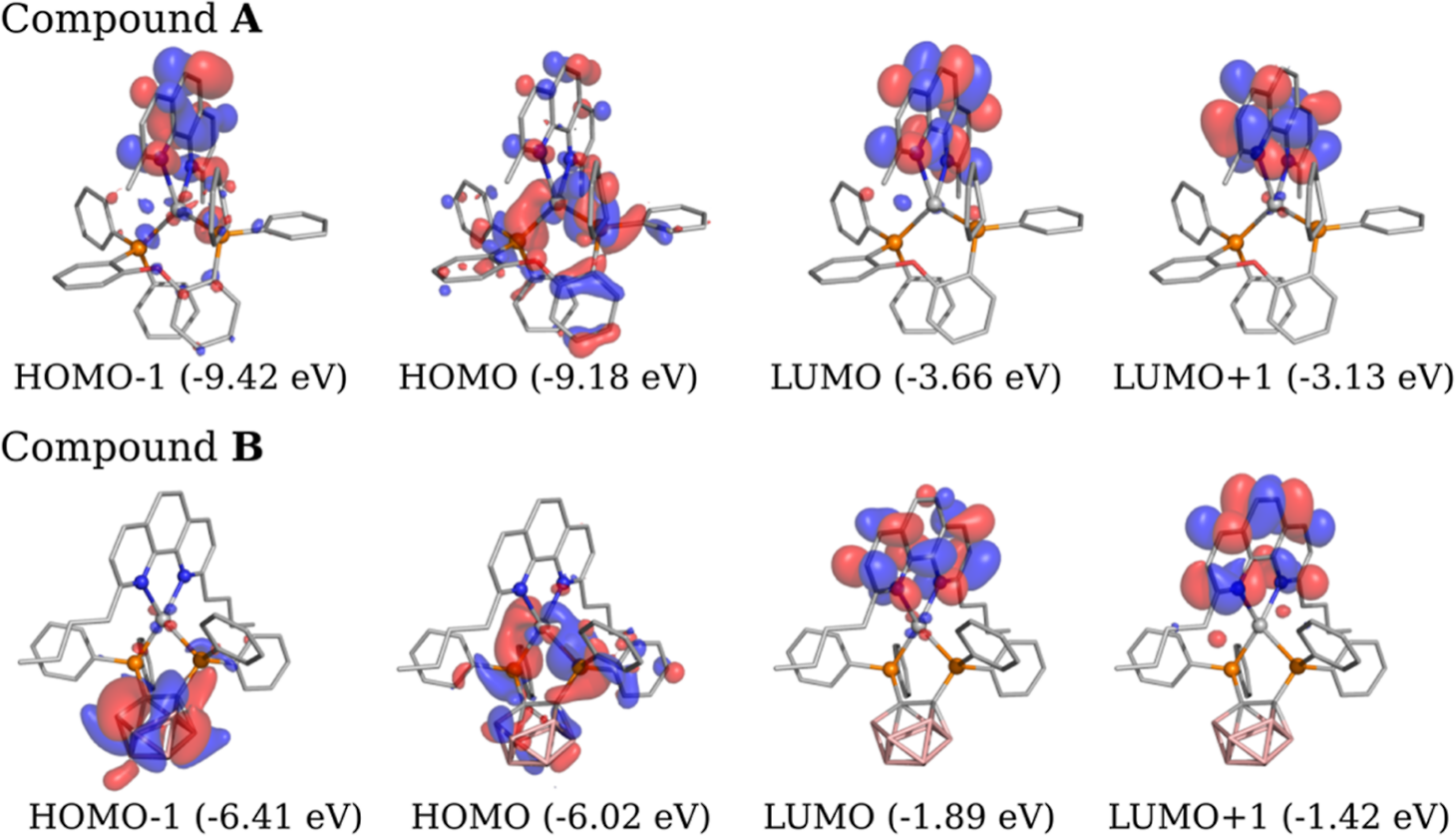
Isosurface contour plots (isovalue = 0.03) of the TD-DFT calculated (M06-2X/def2-TZVP) frontier orbitals of the compounds [Ag­(dmp)­(DPEPhos)]^+^ **A** and Ag­(dbp)­(P_2_-nCB) **B** in the optimized T_1_ state geometry. Color map of the atoms: blue—N, orange—P, red—O, pink—B, gray—Ag, dark gray—C. The hydrogens are omitted for clarity.

The distribution of the molecular orbitals over the compound’s fragments is presented in a Mulliken population analysis (Table 6). For example, for compound **A**, the MOs involved in the S_1_ and T_1_ states are mainly localized in the region of the dmp ligand. Only limited metal d-character is involved in the T_1_ state via the relatively small HOMO → LUMO contribution (of 22%) with HOMO carrying 12% metal character. Therefore, especially, the T_1_ state may roughly be regarded as dmp ligand centered ^3^LC state. Indeed, the d-character is only of minor importance and its impact on the SOC matrix element (SOCME) is weak. Accordingly, the calculated radiative phosphorescence decay time is extremely long amounting to τ_r_(T_1_, cal) = 1660 ms (Table 7 below), while the experimentally determined one is, though still long, significantly shorter amounting to τ_r_(T_1_, exp) = 220 ms (Table 3).

On the other hand, for compound **B**, HOMO–1 and HOMO reside dominantly on the P_2_-nCB ligand, while LUMO is localized on the dmp ligand. Thus, the T_1_ → S_0_ transition, occurring from the relaxed T_1_ state, represents a ligand L′(dmp) to ligand L­(P_2_-nCB) charge transfer (CT) transition. Similarly, the S_1_ → S_0_ transition features the same type of CT transition, as the S_1_ state NTOs in the relaxed S_1_ state geometry (not reproduced) are almost equal to those of the relaxed T_1_ state shown in Figure 11. Further, in compound **B**, the metal contributes by 16% to HOMO (Table 6). In this case, however, the HOMO → LUMO contribution with 92% (S_1_ state) and 90% (T_1_ state), respectively, dominates the lowest excited states (Table 5). Hence, it is justified to assign these states of compound **B** as ^1,3^(L + M)­L’CT states. Due to the higher d-character and thus due to larger SOCME, the experimental radiative phosphorescence decay time of compound **B** with τ_r_(T_1_, exp) = 1570 μs (Table 4) is by a factor of about 140 shorter than the value of 220 ms (Table 3) found for compound **A**. The calculated decay time values follow the same trend, if the two compounds are compared. For completeness, it is noted that, compared to the decay times found for Cu­(I) complexes,^4,6,44^ the radiative T_1_ → S_0_ phosphorescence decay time even of complex **B** may still be regarded as being relatively long.

**Figure 11:**
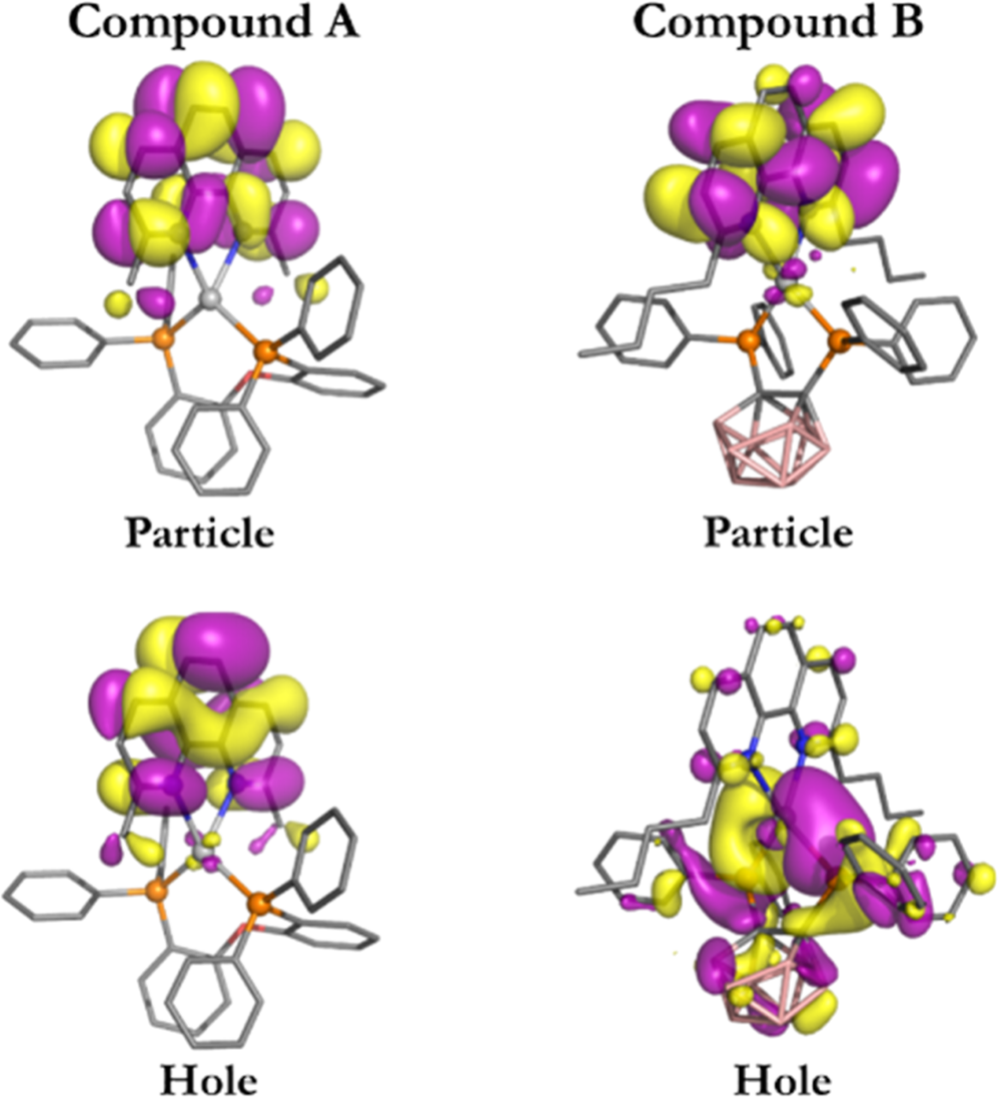
Isosurface contour plots (isovalue = 0.02) of hole and particle natural transition orbitals (NTOs; hole and particle) of the TD-DFT calculated (M06-2X/def2-TZVP) T_1_ state of the compounds [Ag­(dmp)­(DPEPhos)]^+^ **A** and Ag­(dbp)­(P_2_-nCB) **B** in their T_1_ state relaxed geometries. Color map of the atoms: blue—N, orange—P, red—O, pink—B, gray—Ag, dark gray—C. The hydrogens are omitted for clarity.

The pronounced orbital mixtures as calculated for the lowest excited singlet and triplet states recommend use of natural transition orbitals (NTOs) for discussion. The corresponding contour plots for hole and electron orbital distributions in the T_1_ state are reproduced for both compounds in Figure 11. For characterization of phosphorescence transitions, the NTOs are calculated for the optimized T_1_ state geometry. Especially from these plots, it becomes obvious that the T_1_ → S_0_ transitions of compound **A** and **B** can be characterized as ligand-centered (^3^LC → S_0_) and ligand-to-ligand charge transfer transitions with some metal contribution (^3^(L+M)­L’CT → S_0_), respectively.

The NTOs clearly visualize that in compound **A**, the distribution region of the two electrons, one residing in the hole orbital and the other one in the particle orbital, is essentially restricted to the dmp ligand, while in compound **B**, the distribution probability is extended over both ligands (and the metal). Accordingly, the quantum–mechanical exchange interaction that determines the singlet–triplet gap Δ*E*(S_1_–T_1_) is expected to be drastically different for the two compounds. The calculations for the T_1_ state geometry give a ratio of Δ*E*(S_1_–T_1_; **A**)/Δ*E*(S_1_–T_1_; **B**) = 8.94. Experimentally, a ratio of 8.0 is found (Table 7 below). Thus, the estimate, based on the calculated vertical transition energies, describes the trend very well, though the respective values appear markedly overestimated.

It is instructive to shortly discuss changes of S_0_ ↔ S_1_ oscillator strengths *f* that happen with changing the states’ geometry. *f* is proportional to the squared transition dipole moment, to the radiative fluorescence rate, and to the molar extinction coefficient, respectively. As in the excited S_1_ state, significant geometry relaxations occur relative to the electronic ground state geometry, we find distinct energy and oscillator strength differences for the S_0_ → S_1_ transition compared to the S_1_ → S_0_ transition. As seen in Table 7, the *f* values are relatively small for both compounds **A** and **B**. While the S_0_ → S_1_ oscillator strength refers to the molar extinction coefficient measured in absorption, the S_1_ → S_0_ *f* value governs the radiative fluorescence rate *k*_r_(S_1_ → S_0_). Thus, as displayed in eq 2, the TADF decay time is also dominated by the S_1_ → S_0_ oscillator strength. For compound **B**, Table 7 shows that the oscillator strength relevant for the TADF process is by about 40% larger than that determined for the ground state geometry (*f* = 0.071 compared to 0.052 (S_0_ → S_1_)). A comparison for compound **A** is physically not reasonable because we do not observe any fluorescence or TADF from the S_1_ state. Nevertheless, the small *f* value calculated for the S_0_ → S_1_ transition of compound **A** is in line with the small molar extinction coefficient found for the absorption peak at 354 nm of around 10^–3^ M^–1^ cm^–1^ (Figure 3). For completeness, it is noted that the difference between the S_0_ → S_1_ and the S_1_ → S_0_ energies correlates with the Stokes shift between broad band absorption and (if occurring) fluorescence.

In Table 7, a number of calculated photophysical results are summarized and as far as available compared to experimental data. Although we focus on the lowest excited singlet S_1_ and triplet T_1_ states, several higher lying triplets are also listed, as their energies lie between or near to the S_1_ and T_1_ states, though in the optimized ground state geometry. However, in contrast, for the optimized T_1_ state geometry, only one triplet state T_2_ lies between S_1_ and T_1_ for compound **A**, while for compound **B**, no other triplet occurs within the energy gap range. In conclusion, although the calculated energies show some deviation from the experimentally determined ones, trends are displayed well. At this point, it should be taken into account that the calculations are carried out for gas-phase conditions, while the experimental data are determined for the compounds doped in PMMA and imbedded in a rigid crystalline environment, respectively.

Data that relate to dynamic processes are also summarized in Table 7. Interestingly, for compound **A**, the size of the SOCME **⟨**S_1_|H_SO_|T_1_ **⟩** varies by almost 2 orders of magnitude, if the value of the SOCME for the S_1_ state geometry is compared to the one of the T_1_ state optimized geometry. In particular, the theoretical approach gives only a very small value of **⟨**S_1_|H_SO_|T_1_ **⟩** = 0.21 cm^–1^ for the emission process that takes place from the T_1_ state in its relaxed geometry. The squared matrix element enters the radiative rate for the T_1_ → S_0_ emission process (see eq 1). Its small value determines, in part, the very long calculated decay time of 1660 ms. However, if compared to the experimental radiative decay time of 220 ms (Tables 3 and 7), the calculated value is unrealistically long. Inclusion of vibronic coupling processes, not taken into account in our approach, will at least in part shorten the calculated value as by these processes, additional decay paths are opened, enhancing the decay rate^68^ (compare also refs 69 and 70 ). On the other hand, for compound **B** with a significantly larger SOCME of **⟨**S_1_|H_SO_|T_1_ **⟩** = 12.46 cm^–1^ in the relaxed T_1_ state geometry, direct SOC-induced phosphorescence becomes more probable, and thus, vibronic coupling has less impact on the decay rate.^68^ Indeed, the experimental phosphorescence decay time of 1570 μs is only moderately shorter than the calculated one of 2710 μs (Table 7).

The TD-DFT model under inclusion of SOC allows us to calculate also the intersystem crossing rate k­(ISC) from the S_1_ to the T_1_ state. In this case, relaxation takes place from the S_1_ state in its own optimized (relaxed) geometry to the T_1_ state in the same geometry. According to Fermi’s golden rule,^4,55,71^ k­(ISC) is proportional to the squared SOCME. For compound **A**, the SOCME amounts to 14.79 cm^–1^ compared to 8.85 cm^–1^ (compound **B**) (Table 7). Thus, it becomes reasonable that the ISC time of compound **A** calculated to τ­(ISC) = 9 ps is distinctly faster than the one calculated to 20 ps for compound **B**. The ISC times calculated fit well to those observed for Cu­(I) compounds.^3,50,55,72^

The different SOCMEs (0.21 cm^–1^ versus 14.79 cm^–1^, Table 7) of compound **A** govern (among other parameters as displayed in eq 1) crucially the T_1_ → S_0_ phosphorescence and the S_1_ → T_1_ ISC processes, respectively. These dissimilar values are related to the different electronic characters of the states in their relaxed geometries. For phosphorescence from the T_1_ state, the relaxed T_1_ state geometry is responsible. The corresponding NTOs, displayed in Figure 11, are largely ligand centered of HOMO–1 → LUMO character and involve only small 4d-orbital character. Hence, SOC between the T_1_ and S_1_ states is rather weak (0.21 cm^–1^). On the other hand, the ISC process from the S_1_ state to the T_1_ state takes place from the S_1_ state in its relaxed geometry. In this situation, however, the state’s character is drastically altered. Both S_1_ and T_1_ states are now essentially of charge transfer character. This is shown in Figure 12, where we reproduce the calculated NTOs of the S_1_ and T_1_ states of compound **A** optimized for the S_1_ state geometry. The CT character and, in particular, a larger involvement of d-orbitals become obvious. Hence, an almost 2 orders of magnitude larger value of **⟨**S_1_|H_SO_|T_1_ **⟩** = 14.79 cm^–1^ results. And this leads to relatively fast ISC in compound **A**. For completeness, it is noted that for compound **B**, the corresponding NTOs do not indicate strong differences of d-orbital contribution in the different geometries. Thus, the SOMEs calculated (8.85 cm^–1^ versus 12.46 cm^–1^) for the different geometries of **B** do not deviate significantly (Table 7).

**Figure 12:**
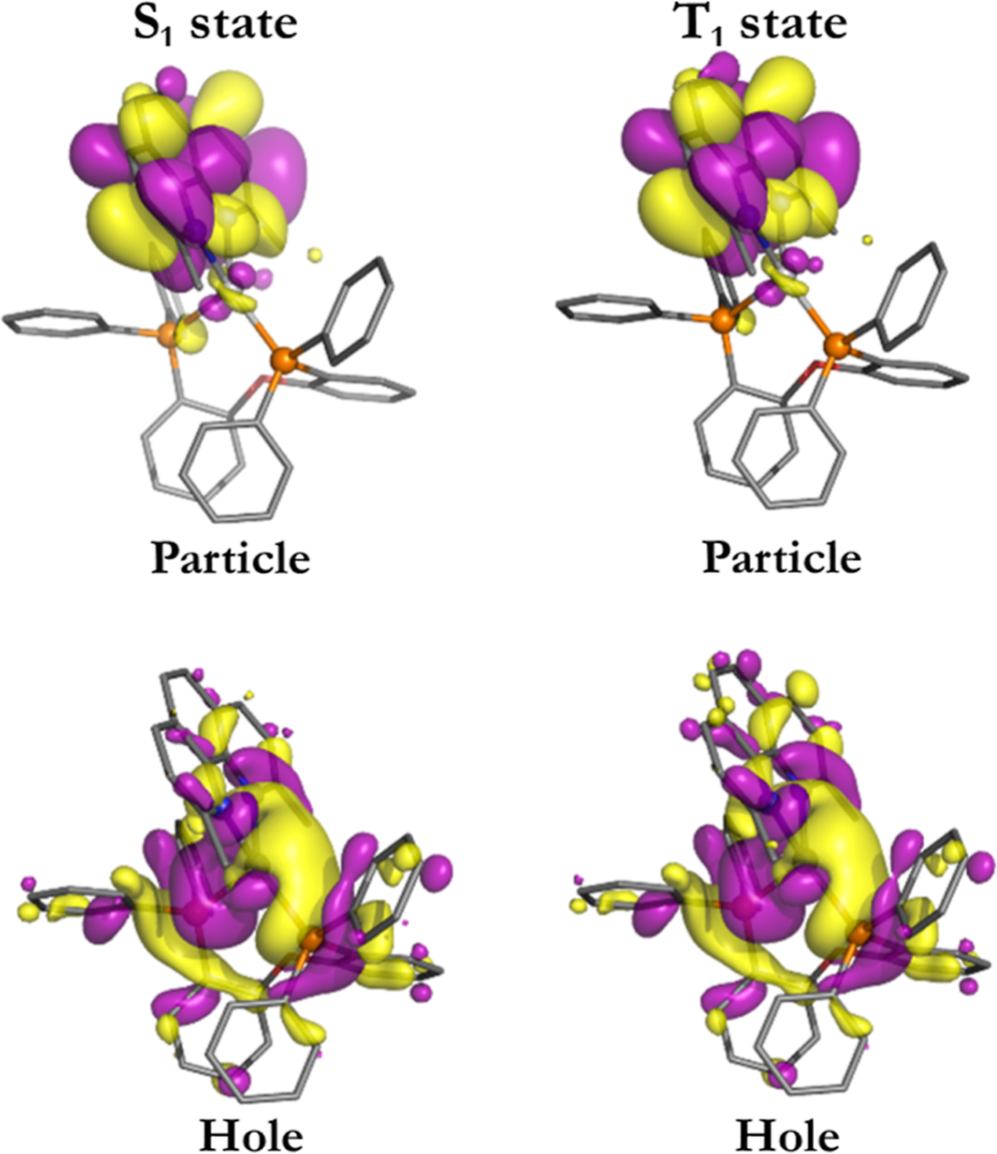
Isosurface contour plots (isovalue = 0.02) of hole and particle natural transition orbitals (NTOs) of the TDDFT-calculated (M06-2X/def2-TZVP) T_1_ and S_1_ states of [Ag­(dmp)­(DPEPhos)]^+^ **A** in the optimized S_1_ state geometry. Color map of the atoms: blue—N, orange—P, red—O, pink—B, gray—Ag, and dark gray—C. The hydrogens are omitted for clarity.

### 6.3 Modification of Phenanthroline Substitution

Finally, we study theoretically the impact of replacement of the dmp (2,9-dimethyl-1,10-phenanthroline) ligand of compound **A** by dbp (2,9-di-*n*-butyl-1,10-phenanthroline). This is a pronounced sterically demanding substitution. The calculations show that this replacement results only in a small red shift of the T_1_ state energy by 0.02 eV in the T_1_ state optimized geometry and in stabilization of the frontier orbital energies (HOMO–1, HOMO, LUMO, and LUMO+1) by around 0.1 eV. Apart from small geometry changes, the orbitals are largely similar. Thus, the character of the T_1_ state is not altered. However, as the replacement of dmp with dbp leads to a stiffer compound, a moderate increase of photoluminescence quantum yield is expected to occur. On the contrary, replacement of dbp by dmp in Ag­(dbp)­(P_2_-nCB) (compound **B** = compound **24** in Table 2) yielding compound **23** shows distinct changes of the emission properties. The TADF emission quantum yield at *T* = 300 K is reduced from Φ_PL_ = 100% to 78% (Table 2) by this replacement mainly due to the lower internal rigidity of Ag­(dmp)­(P_2_-nCB) (compound **23**) compared to **B**. Moreover, the radiative S_1_ → S_0_ fluorescence rate decreases from 5.6 × 10^7^ s^–1^ (formally 18 ns) (compound **B**, Table 4) to 2.8 × 10^7^ s^–1^ (formally 36 ns) (dmp compound **23**, Table 2).^41^ Clearly, this type of ligand modification, giving a less rigid complex, allows pronounced geometry changes upon ligand-to-ligand charge transfer transition. Obviously, in this case, a distinct influence on the material’s photophysical properties is occurring. For detailed discussion, it is referred to ref 41.

## 7 Concluding Summary

Recently, interest in photophysical especially photoluminescence properties of Ag­(I) compounds is strongly growing. On the one hand, this is related to occurrence of bright long-lived phosphorescence and, on the other hand, to appearance of efficient and short-lived thermally activated delayed fluorescence (TADF). In particular, in recent years, an increasing number of Ag­(I)-TADF compounds have been reported, stimulated by potential application as OLED emitters. In this mini-review, we present several examples of both types of compounds. Assignment of the respective emission characteristics is often not straightforward. Thus, we developed criteria to characterize the transition type.

Ambient-temperature phosphorescence of Ag­(I) compounds is usually based on ligand-centered transitions of the ^3^LC → S_0_ type, whereby the related singlet state S_1_ state lies too high in energy (several thousand wavenumbers, several 100 meV) to be efficiently thermally accessible at ambient temperature. Frequently, the emission resembles that of organic molecules showing ^3^ππ* → S_0_ transitions, though usually some Ag­(I) 4d-orbital admixture leads to increased SOC to higher-lying singlet states and thus to higher allowedness of the T_1_ → S_0_ transition compared to that of purely organic molecules. Accordingly, the Ag­(I) compounds can exhibit bright ambient-temperature phosphorescence with emission decay time in the range from 1 to more than 100 ms. Compound **A** represents an exemplary material showing bright and exceptionally long-lived phosphorescence at ambient temperature.

TADF is found for Ag­(I) compounds that exhibit charge transfer (CT) character of the lowest excited states, as in this case, the important requirement for small energy gap Δ*E*(S_1_–T_1_) of around thousand wavenumbers (≈120 meV) or smaller is easily realizable. Interestingly, different chemical design strategies can lead to compounds with low-energy ^1,3^CT states at small energy gap, such as compounds exhibiting (a) intraligand CT transitions giving ^1,3^ILCT states, (b) ligand-to-ligand’ CT transitions resulting in ^1,3^LL’CT states, and (c) metal-to-ligand CT transitions providing ^1,3^MLCT states, respectively. Frequently, ^1,3^MLCT states experience additional X character induced by coordinated halides X or similar groups. In this case, ^1,3^(M+X)­LCT states are resulting. For completeness, it is noted that mostly more metal Ag­(I)-4d-orbital character is involved in the ^1,3^LL’CT states, giving ^1,3^(L+M)­L’CT states, than in states of ^1,3^ILCT character. This is important, as the 4d-admixture induces increased SOC and thus leads to shorter ISC, RISC, and phosphorescence time constants.

Usually, due to the CT character of the excited states, significant geometry changes occur upon excitation. Hence, excited state energies are stabilized, and thus, the resonance condition for energy transfer is not fulfilled, or in other words, the excitation happens to be self-trapped. As a consequence, frequently concentration quenching is not effective for transition-metal–organic TADF compounds. Therefore, high emitter concentrations or even neat materials exhibit bright TADF. This is in contrast to the situation frequently found for compounds that feature ^3^LC → S_0_ phosphorescence; they often show efficient concentration quenching.

Among the Ag­(I) complexes presented, two highlights with respect to their photophysical properties become obvious: [Ag­(dmp)­(DPEPhos)]^+^, cation of compound **A**, is an outstanding material, as it shows bright ^3^LC → S_0_ photoluminescence with an extraordinarily long ambient-temperature emission decay time of τ = 110 ms at Φ_PL_ = 50%. On the other hand, Ag­(dbp)­(P_2_-nCB), compound **B**, represents a breakthrough TADF compound. It exhibits the shortest ambient-temperature TADF decay time of τ = 1.4 μs at Φ_PL_ = 100% reported for pseudotetrahedrally coordinated compounds, so far. As compound **A** represents a very sensitive luminescent oxygen sensor material and compound **B** potentially constitutes a very efficient OLED emitter material, we characterize both compounds in detail. In particular, we study steady-state emission and emission dynamics properties down to cryogenic temperatures and investigate related properties by DFT and TD-DFT methods, including SOC that allows us to characterize both LC and CT transitions.

The calculations display that for both compounds, the orbitals involved in the lowest excited triplet states are based on different orbital components, in particular, mainly on HOMO–1 → LUMO and HOMO → LUMO contributions. Thus, for example, the T_1_ state in its optimized geometry has to be described by natural transition orbitals (NTOs). Indeed, these display the T_1_ state character clearly as ligand-centered ^3^LC type for compound **A** and as ligand-to-ligand charge transfer ^3^(L + M)­L’CT type (showing some Ag 4d-orbital admixture) for compound **B**. Moreover, trends found from experimental studies, such as variation of the energy gap and emission decay time, are displayed by the calculations, although the theoretical values show some deviation from the experimental ones. Moreover, ISC times calculated to 9 ps (compound **A**) and to 20 ps (compound **B**) fit well to the range of values reported for Cu­(I) complexes.

Briefly, in this mini-review, we present a large number of organic-Ag­(I) compounds that show ambient-temperature phosphorescence or TADF. We highlight two compounds by detailed photoluminescence and theoretical DFT and TD-DFT/SOC studies. [Ag­(dmp)­(DPEPhos)]^+^, complex **A**, represents an outstanding material, as it shows extremely long phosphorescence decay time of τ = 110 ms at Φ_PL_ = 50%, while Ag­(dbp)­(P_2_-nCB), compound **B**, being also a breakthrough material, exhibits around a 5 orders of magnitude shorter TADF decay time of τ = 1.4 μs at Φ_PL_ = 100%, properties not reported for any other tetrahedrally coordinated compound, so far. Potentially, both compounds may be applied as a very sensitive optical oxygen sensor material or as an efficient OLED emitter material, respectively.

## Data Availability

Data will be made available on request.
